# Comparative effectiveness of pharmacological and non-pharmacological interventions for dyspnea management in advanced cancer: A systematic review and network meta-analysis

**DOI:** 10.1016/j.apjon.2025.100671

**Published:** 2025-02-18

**Authors:** An Thuy Vo, Kim-Ngan Thi Ta, Kai-Jen Chuang

**Affiliations:** a Faculty of Public Health, University of Medicine and Pharmacy at Ho Chi Minh City, Ho Chi Minh, Viet Nam; b Department of Public Health, School of Medicine, College of Medicine, Taipei Medical University, New Taipei, Taiwan; c School of Public Health, College of Public Health, Taipei Medical University, New Taipei, Taiwan

**Keywords:** Dyspnea, Advanced cancer, Palliative care, Network meta-analysis, Breathlessness

## Abstract

**Objective:**

This study aimed to evaluate and rank the effectiveness of pharmacological and non-pharmacological interventions for managing dyspnea severity, anxiety, exercise capacity, and health-related quality of life (HRQoL) in patients with advanced cancer.

**Methods:**

A comprehensive search of PUBMED, HINARI, CENTRAL, and ResearchGate was conducted to identify randomized controlled trials (RCTs) published up to March 2024. Network meta-analysis was performed to compare interventions, calculating mean differences (MD) and standardized mean differences (SMD) with 95% confidence intervals (CI). P-scores were used to rank the interventions. Risk of bias was assessed using the Cochrane tool, and the quality of evidence (QOE) was evaluated using the GRADE framework.

**Results:**

A total of 42 RCTs, encompassing 3,832 patients, were included in the analysis. Among the evaluated interventions, high-flow nasal cannula (HFNC) demonstrated the most significant improvement in dyspnea relief (SMD = −1.91; 95% CI: −3.32 to −0.49; QOE: moderate), followed by acupressure/reflexology (SMD = −1.04; 95% CI: −2.02 to −0.06; QOE: very low). Activity rehabilitation was the only intervention that significantly reduced anxiety compared to the control group (SMD = −0.64; 95% CI: −0.97 to −0.32; QOE: very low). While all interventions showed trends of improving exercise capacity, none reached statistical significance. Notably, acupressure/reflexology significantly enhanced HRQoL (SMD = 1.55; 95% CI: 0.22 to 2.88; QOE: moderate).

**Conclusions:**

Non-pharmacological interventions, particularly HFNC and acupressure/reflexology, were more effective than pharmacological approaches in improving dyspnea relief and HRQoL. However, the low quality of evidence underscores the need for high-quality, large-scale trials to confirm these findings and refine treatment strategies for dyspnea management in advanced cancer patients.

**Systematic review registration:**

PROSPERO CRD42023479041.

## Introduction

Dyspnea, commonly known as “shortness of breath” or “breathlessness”, is one of the most distressing symptoms experienced by patients who have advanced cancer.^1^ This syndrome not only makes breathing difficult but also affects daily life in many ways. It can cause anxiety, limit physical activity, and reduce overall well-being. A previous research has shown that advanced cancer patients with dyspnea often struggle with daily activities, making even simple tasks challenging.^2^ These limitations lead to reduced social functioning of patients and make them more dependent on caregivers, which in turn exacerbates their psychological distress. Therefore, effective management of dyspnea is essential in palliative care for patients with advanced cancer.

Many interventions have been used to alleviate dyspnea in these patients, ranging from pharmacological interventions to non-pharmacological interventions. In particular, pharmacological treatments primarily aim to target underlying physiological issues or provide symptomatic relief.^3^ This approach may involve directly addressing respiratory symptoms or alleviating associated conditions, such as inflammation.^4^ On the other hand, non-pharmacological methods often focus on enhancing comfort, functionality, and overall well-being without medication.^4^ However, the effectiveness of these interventions varies widely, and there is no consensus on the optimal treatment for managing dyspnea across multiple outcomes, including dyspnea severity, anxiety, exercise capacity, and health-related quality of life (HRQoL). Furthermore, existing studies often focused on individual interventions or limited outcomes.^5^, ^6^, ^7^, ^8^ This narrow focus overlooks the complex and multifaceted nature of dyspnea in palliative care, where a more holistic evaluation is essential. Additionally, some prior researches have become less relevant due to advancements in clinical practices and the introduction of new interventions.^9^^,^^10^ These gaps in the literature highlight the need for a comprehensive comparative analysis that simultaneously compares and ranks both pharmacological and non-pharmacological interventions across several outcomes.

This study aims to address these gaps by conducting a network meta-analysis to evaluate and rank the effectiveness of various interventions for dyspnea management in advanced cancer patients. By assessing four outcomes: dyspnea severity, anxiety, exercise capacity, and HRQoL; this analysis provides a comprehensive understanding of how each intervention affects key aspects of patient well-being. The findings from this study will offer valuable guidance on selecting the most effective strategies, contributing to improved symptom management and palliative care to this vulnerable population.

## Methods

This systematic review and network meta-analysis adhered to the PRISMA Extension Statement for Reporting of Systematic Reviews Incorporating Network Meta-analyses of Health Care Interventions (PRISMA-NMA).^11^

### Search strategy

A comprehensive literature search was conducted until March 2024 through four databases: PUBMED, CENTRAL, HINARI, and ResearchGate. The search terms included combinations of “advanced cancer”, “dyspnea”, “intervention”, and “random” with the detailed strategy provided in Appendix 1. To ensure thoroughness, we also manually reviewed the bibliographies of relevant studies and previously published meta-analyses, employing a snowballing technique to identify additional eligible studies.

The eligibility criteria were defined as follows: (1) Randomized controlled trials (RCTs) assessing interventions for relieving symptom of dyspnea in adult patients (aged 18 years and older) diagnosed with advanced-stage cancer; (2) The control groups were defined as either placebo, usual care, sham interventions, or any form of general practitioner care referred to as treatment as usual; (3) Studies had to report alleviation in dyspnea severity as the outcome, measured by validated dyspnea assessment tools. Additionally, only articles written in the English language were included. Articles that included multiple intervention groups but lacked a control group for comparison were also excluded. Trials with the proportion of cancer patients less than 50% of the total population were excluded.^9^^,^^10^

### Study selection and data extraction

The first author reviewed titles and abstracts to assess relevance and retrieved full-text articles for further evaluation. Each full-text article was rigorously assessed against our predefined inclusion and exclusion criteria. The second author checked these results.

Articles were excluded if no response was received or if the required data remained unavailable. Moreover, any disagreements during the selection process were resolved through consensus or by consulting with the third researcher. The whole process was illustrated using the PRISMA flowchart 2020.^12^

The first author extracted and categorized detailed characteristics of these trials by intervention types, first author, publication year, study design, patient characteristics, sample size, intervention details, control group, follow-up duration, outcome variables and post-intervention outcome data (including the mean scores and standard deviations) directly from each RCT. Additionally, outcomes were extracted at the time of assessment using Intention-to-Treat (ITT) analysis technique. In circumstances where authors utilized per-protocol (PP) analysis or did not specify their technique, we would use the available reporting data.

In cases where the data is represented in graphs, the WebPlotDigitizer tool was employed for data extraction to estimate the outcomes.^13^ To address any missing data, we contacted the corresponding authors, allowing a two-week response window. The second author supervised and checked the process of data extraction. Again, any disagreements were solved through consensus or by consulting with the third author.

#### Outcomes and summary measures

Our study focused on several key outcome variables. The primary outcome was dyspnea severity, measured as a continuous variable using validated assessment scales to quantify the level or intensity of breathlessness experienced by advanced cancer patients. When studies used multiple scales to quantify dyspnea severity, the primary outcome measure reported was extracted. If scales were not distinctly prioritized, data were extracted in the following order of priority, which based on the frequency of reporting in the included RCTs: (1) Numeric Rating Scale (NRS), (2) Visual Analog Scale (VAS), (3) Borg scale, (4) Dyspnoea-12, and (5) other scales.

We also examined three secondary outcomes, all of which are continuous variables: (1) Anxiety, measured using multi-item emotional response scales; (2) Exercise capacity, also known as exercise tolerance, evaluated using the 6-Minute Walk Test (6MWT) to measure the maximum physical exertion a patient can sustain; and (4) Health-related quality of life (HRQoL), assessing the overall impact of dyspnea and its treatment on patients' well-being, measured by the global or general health or mastery score related to HRQoL scales.

In our study, we processed continuous outcome data using several methods. Firstly, we calculated the Mean Difference (MD) for continuous outcomes reported on the same scale, and the Standardized Mean Difference (SMD) for data reported on different scales, all results presented with 95% confidence intervals (CIs). Moreover, we followed Chapter 6 of the Cochrane Handbook to calculate standard deviations (SD) from standard errors (SE) and CIs, obtain SDs for mean differences, and combine groups.^14^ For studies reporting interquartile ranges, we used formulas by Wan et al. to estimate means and SDs.^15^ Similarly, we followed methods proposed by Hozo et al. to convert median and range to mean and standard deviation.^16^

#### Classification of the intervention and control groups

Based on the classification of previous meta-analysis,^9^^,^^10^ the intervention groups include: (1) Airflow; (2) High-flow nasal cannula (HFNC); (3) Bilevel ventilation; (4) Compressed air; (5) Behavioral psychoeducational interventions; (6) Activity and rehabilitation interventions; (7) Acupuncture; (8) Acupressure or Reflexology; (9) Lay foot manipulation; (10) Activity rehabilitation ​+ ​Behavioral psychoeducational (AR ​+ ​BP); (11) Behavioral psychoeducational ​+ ​Integrative medicine (BP ​+ ​IM); (12) Activity rehabilitation ​+ ​Behavioral psychoeducational ​+ ​Integrative medicine (AR ​+ ​BP ​+ ​IM); (13) Opioids; (14) Corticosteroids; (15) Anxiolytics; (16) Cannabidiol.

The control groups involve some forms of usual care referred to as treatment as usual, standard care, sham control, fan-to-legs, standard supplemental oxygen,^8^^,^^17^ conventional medical follow-up, nebulized saline,^18^ or placebo.

### Data analysis

We performed all data analyses using the “netmeta” package in R software, version 4.2.2.^19^^,^^20^ To visualize the network and connection nodes,^21^ we plotted network graphs for each outcome variable. The effectiveness of interventions was ranked using P-scores, which range from 0 (indicating the lowest effectiveness) to 1 (indicating the highest effectiveness).^22^

We used τ^2^ and I^2^ statistics to assess network heterogeneity for all treatment comparisons. The I^2^ statistic was interpreted with the following thresholds: 0%–40% as potentially unimportant, 30%–60% as moderate, 50%–90% as substantial, and 75%–100% as considerable heterogeneity.^23^ We used the Q statistic to evaluate global inconsistency within a design-by-treatment model with random effects.^24^ Local inconsistency was assessed using a node split method and a back-calculation approach.^25^ For all analyses, we considered *P* values less than 0.05 to be statistically significant. We also conducted sensitivity analyses for all outcomes to evaluate the robustness of the results by excluding studies that showed a high risk of bias of overall domains. Ultimately, we generated comparison-adjusted funnel plots and Egger's test for outcomes with at least 10 RCTs to investigate potential publication bias.^26^^,^^27^

### Risk of bias (ROB) and quality of evidence assessments

We appraised the ROB for each RCT and crossover RCT using the Cochrane Risk of Bias version 2 tool (ROB 2.0).^28^ In general, appraisals were guided by the tool, with any discrepancies between two assessors and the algorithm reviewed by the third author in the research team. The results were summarized using both summary plots and traffic light plots, which were generated using the Shiny app.^29^^,^^30^ Furthermore, we used the Grading of Recommendations, Assessment, Development, and Evaluation (GRADE) tool to assess the quality of evidence (QOE) across studies for each outcome.^31^

## Results

### Study selection

**Fig. 1:**
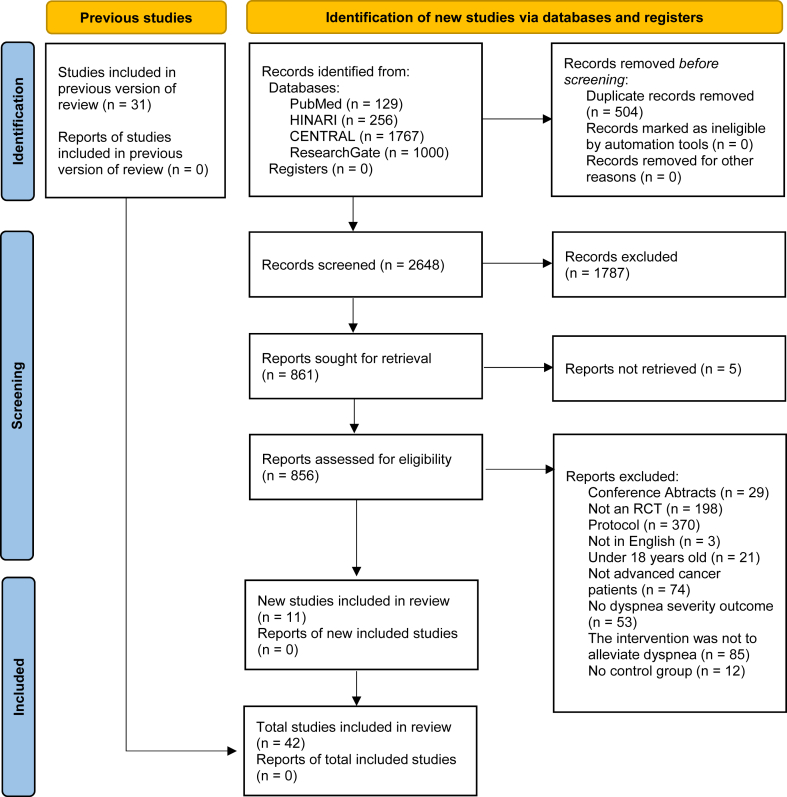
PRISMA flowchart.

The initial search of electronic databases included studies published until March 2024, resulting in 3152 research articles being identified from four databases. Of these, 856 articles were appropriate for full-text review. 42 research articles were included in this network meta-analysis. These studies also encompass those identified from previous meta-analyses conducted before the search period (Fig. 1).

### Characteristics of included studies

A total of 3832 patients were enrolled in this network meta-analysis. These included 10 crossover RCTs and 32 RCTs. The publication dates of these studies ranged from the oldest in 1993 to the most recent in 2023. Each RCT varied in size, with participant numbers ranging from 9 to 379 individuals. Breast and lung cancer were the most reported types of cancer among the study populations. Among 42 RCTs, 11 RCTs focused on pharmacological interventions. The longest follow-up duration was one year. Detailed characteristics of each RCT are presented in Appendix 2.

### Network meta-analysis

#### Network plots

For managing dyspnea, the network of intervention comparisons consisted of 16 nodes among 42 trials (Appendix 3). With direct links to every other intervention, the control group was the most interconnected intervention. Only one trial^32^ had three eligible groups, and the other trials^6^^,^^33^, ^34^, ^35^, ^36^, ^37^, ^38^, ^39^, ^40^, ^41^, ^42^, ^43^, ^44^, ^45^, ^46^, ^47^, ^48^, ^49^, ^50^, ^51^, ^52^, ^53^, ^54^, ^55^, ^56^, ^57^, ^58^, ^59^, ^60^, ^61^, ^62^, ^63^, ^64^, ^65^, ^66^, ^67^, ^68^, ^69^, ^70^, ^71^, ^72^ had two eligible groups.

For anxiety, the network of intervention comparisons consisted of 9 individual nodes among 11 trials (Appendix 3). Only one trial^32^ had three eligible groups, and the other trials^6^^,^^38^^,^^39^^,^^41^, ^42^, ^43^^,^^45^^,^^59^^,^^60^^,^^63^ had two eligible groups.

For exercise capacity, the network of intervention comparisons consisted of 6 individual nodes among 11 trials (Appendix 3). All trials^33^^,^^37^^,^^39^^,^^51^^,^^54^^,^^56^^,^^58^^,^^61^^,^^70^, ^71^, ^72^ had 2 eligible groups.

For health-related quality of life, the network of intervention comparisons consisted of 9 individual nodes among 14 trials (Appendix 3). Only one trial^32^ had three eligible groups, and the other trials^35^^,^^37^^,^^39^, ^40^, ^41^^,^^44^^,^^46^^,^^47^^,^^53^^,^^54^^,^^56^^,^^59^^,^^64^ had two eligible groups.

#### Efficacy outcomes and rank probabilities

Pairwise meta-analyses and Network meta-analyses of the overall effect for all outcomes are shown in Table 1 and the ranking of the effectiveness of interventions based on the P-score are in Table 2. The global and local inconsistency of all outcomes are included in Appendix 4 and 5. The QOE for each comparison is in Appendix 6 and 7.

a) Dyspnea severity

**Table 1:** Pairwise meta-analysis estimates and network meta-analysis estimates of outcomes: a) Dyspnea Severity, b) Anxiety, c) Exercise Capacity, d) Health-related Quality of Life.

*a) Dyspnea severity*																
**HFNC**													**−1.91 (−3.32; −0.49)**			
−0.87 (−2.59; 0.86)	**Acupressure/Reflexology**					0.00 (−1.32; 1.32)							**−1.04 (−2.02; -0.06)**			
−1.09 (−2.73; 0.54)	−0.23 (−1.51; 1.05)	**Airflow**											−0.81 (−1.63; 0.01)			
−1.42 (−2.95; 0.12)	−0.55 (−1.69; 0.59)	−0.32 (−1.33; 0.69)	**Opioids**										−0.49 (−1.08; 0.10)			
−1.49 (−2.98; 0.00)	−0.62 (−1.72; 0.47)	−0.40 (−1.35; 0.55)	−0.07 (−0.83; 0.69)	**Activity rehabilitation**									−0.42 (−0.90; 0.06)			
−1.48 (−3.42; 0.45)	−0.62 (−2.26; 1.02)	−0.39 (−1.94; 1.16)	−0.07 (−1.51; 1.38)	0.01 (−1.40; 1.41)	**Bilevel ventilation**								−0.42 (−1.74; 0.89)			
−1.56 (−3.45; 0.32)	−0.70 (−1.94; 0.55)	−0.47 (−1.96; 1.02)	−0.15 (−1.52; 1.23)	−0.07 (−1.41; 1.26)	−0.08 (−1.89; 1.73)	**Lay foot manipu-lation**							0.36 (−0.96; 1.68)			
−1.61 (−3.23; 0.02)	−0.74 (−2.00; 0.52)	−0.51 (−1.65; 0.63)	−0.19 (−1.18; 0.80)	−0.12 (−1.05; 0.81)	−0.12 (−1.66; 1.42)	−0.04 (−1.52; 1.44)	**AR ​+ ​BP**						−0.30 (−1.10; 0.50)			
**−1.63 (−3.17; −0.09)**	−0.76 (−1.91; 0.39)	−0.53 (−1.55; 0.48)	−0.21 (−1.06; 0.63)	−0.14 (−0.91; 0.63)	−0.15 (−1.60; 1.30)	−0.07 (−1.45; 1.32)	−0.02 (−1.02; 0.98)	**AR ​+ ​BP** **+IM**					−0.28 (−0.88; 0.33)			
−1.72 (−3.45; 0.00)	−0.86 (−2.24; 0.53)	−0.63 (−1.91; 0.65)	−0.31 (−1.45; 0.83)	−0.23 (−1.33; 0.86)	−0.24 (−1.88; 1.40)	−0.16 (−1.75; 1.42)	−0.12 (−1.38; 1.14)	−0.10 (−1.25; 1.05)	**Behavioral psycho educational**				−0.18 (−1.16; 0.80)			
**−1.80 (−3.53; −0.07)**	−0.93 (−2.33; 0.46)	−0.71 (−2.00; 0.59)	−0.38 (−1.54; 0.78)	−0.31 (−1.42; 0.80)	−0.32 (−1.97; 1.34)	−0.24 (−1.83; 1.36)	−0.19 (−1.47; 1.08)	−0.17 (−1.34; 0.99)	−0.07 (−1.47; 1.32)	**Corti-costeroids**			−0.11 (−1.10; 0.89)			
−1.89 (−3.84; 0.06)	−1.03 (−2.69; 0.64)	−0.80 (−2.37; 0.78)	−0.48 (−1.94; 0.99)	−0.40 (−1.83; 1.03)	−0.41 (−2.29; 1.47)	−0.33 (−2.16; 1.50)	−0.29 (−1.85; 1.28)	−0.26 (−1.74; 1.21)	−0.17 (−1.83; 1.50)	−0.09 (−1.77; 1.58)	**Cann-abidiol**		−0.01 (−1.36; 1.33)			
**−2.03 (−3.95; −0.11)**	−1.17 (−2.79; 0.46)	−0.94 (−2.47; 0.60)	−0.62 (−2.04; 0.81)	−0.54 (−1.92; 0.84)	−0.55 (−2.40; 1.30)	−0.47 (−2.27; 1.33)	−0.43 (−1.95; 1.09)	−0.40 (−1.83; 1.03)	−0.31 (−1.93; 1.32)	−0.23 (−1.87; 1.40)	−0.14 (−2.01; 1.73)	**Anxio-lytics**	0.13 (−1.17; 1.42)			
**−1.91 (−3.32; −0.49)**	**−1.04 (−2.02; −0.06)**	−0.81 (−1.63; 0.01)	−0.49 (−1.08; 0.10)	−0.42 (−0.90; 0.06)	−0.42 (−1.74; 0.89)	−0.34 (−1.59; 0.90)	−0.30 (−1.10; 0.50)	−0.28 (−0.88; 0.33)	−0.18 (−1.16; 0.80)	−0.11 (−1.10; 0.89)	−0.01 (−1.36; 1.33)	0.13 (−1.17; 1.42)	**Control group**	−0.29 (−1.10; 0.52)	−0.54 (−1.97; 0.89)	−0.58 (−2.05; 0.89)
**−2.20 (−3.83; −0.57)**	**−1.33 (−2.60; −0.06)**	−1.10 (−2.25; 0.05)	−0.78 (−1.78; 0.22)	−0.71 (−1.65; 0.23)	−0.72 (−2.26; 0.83)	−0.63 (−2.12; 0.85)	−0.59 (−1.72; 0.54)	−0.57 (−1.58; 0.44)	−0.47 (−1.74; 0.80)	−0.40 (−1.68; 0.88)	−0.31 (−1.87; 1.26)	−0.17 (−1.69; 1.36)	−0.29 (−1.10; 0.52)	**Compressed air**		
**−2.44 (−4.46; −0.43)**	−1.58 (−3.31; 0.16)	−1.35 (−3.00; 0.30)	−1.03 (−2.58; 0.52)	−0.95 (−2.46; 0.56)	−0.96 (−2.91; 0.98)	−0.88 (−2.78; 1.02)	−0.84 (−2.48; 0.80)	−0.82 (−2.37; 0.74)	−0.72 (−2.45; 1.02)	−0.64 (−2.39; 1.10)	−0.55 (−2.52; 1.41)	−0.41 (−2.34; 1.52)	−0.54 (−1.97; 0.89)	−0.25 (−1.89; 1.40)	**BP ​+ ​IM**	
**−2.48 (−4.53; −0.44)**	−1.62 (−3.39; 0.15)	−1.39 (−3.07; 0.29)	−1.07 (−2.65; 0.52)	−0.99 (−2.54; 0.55)	−1.00 (−2.98; 0.97)	−0.92 (−2.85; 1.01)	−0.88 (−2.55; 0.79)	−0.86 (−2.45; 0.73)	−0.76 (−2.53; 1.01)	−0.68 (−2.46; 1.09)	−0.59 (−2.59; 1.40)	−0.45 (−2.41; 1.51)	−0.58 (−2.05; 0.89)	−0.29 (−1.96; 1.39)	−0.04 (−2.09; 2.01)	**Acu-puncture**


b) Anxiety								
**Activity rehabilitation**					**−0.64 (−0.97; −0.32)**			
−0.25 (−0.99;c0.49)	**Behavioral psychoeducational + integrative medicine**				−0.40 (−1.06; 0.27)			
**−0.53 (−0.94; −0.12)**	−0.28 (−0.99; 0.43)	**Activity rehabilitation + Behavioral psychoeducational + integrative medicine**			−0.12 (−0.36; 0.13)			
**−0.57 (−1.08; −0.07)**	−0.32 (−1.10; 0.45)	−0.05 (−0.50; 0.41)	**Lay foot manipulation**		−0.07 (−0.46; 0.32)		−0.19 (−0.56; 0.19)	
**−0.64 (−1.11; −0.17)**	−0.39 (−1.14; 0.35)	−0.11 (−0.53; 0.30)	−0.07 (−0.58; 0.45)	**Activity rehabilitation ​+ Behavioral psycho educational**	−0.00 (−0.34; 0.33)			
**−0.64 (−0.97; −0.32)**	−0.40 (−1.06; 0.27)	−0.12 (−0.36; 0.13)	−0.07 (−0.46; 0.32)	−0.00 (−0.34; 0.33)	**Control group**	−0.13 (−0.77; 0.52)	−0.12 (−0.50; 0.27)	−0.15 (−0.41; 0.12)
**−0.77 (−1.50; −0.05)**	−0.52 (−1.45; 0.41)	−0.24 (−0.93; 0.45)	−0.20 (−0.95; 0.55)	−0.13 (−0.86; 0.60)	−0.13 (−0.77; 0.52)	**Airflow**		
**−0.76 (−1.26; −0.26)**	−0.51 (−1.28; 0.26)	−0.23 (−0.69; 0.22)	−0.19 (−0.56; 0.19)	−0.12 (−0.63; 0.39)	−0.12 (−0.50; 0.27)	0.01 (−0.74; 0.76)	**Acupressure/ Reflexology**	
**−0.79 (−1.21; −0.37)**	−0.54 (−1.26; 0.18)	−0.26 (−0.62; 0.10)	−0.22 (−0.69; 0.25)	−0.15 (−0.58; 0.28)	−0.15 (−0.41; 0.12)	−0.02 (−0.72; 0.68)	−0.03 (−0.50; 0.44)	**Anxiolytics**

c) Exercise capacity					
**Activity rehabilitation**					**56.10 (−16.96; 129.16)**
8.77 (−133.61; 151.15)	**Acupressure or Reflexology**				47.33 (−74.88; 169.54)
48.80 (−193.27; 290.87)	40.03 (−221.11; 301.17)	**Activity rehabilitation** **+Behavioral psychoeducational**			7.30 (−223.48; 238.08)
49.49 (−53.78; 152.77)	40.72 (−101.63; 183.08)	0.69 (−241.36; 242.75)	**Opioids**		6.61 (−66.39; 79.61)
57.01 (−89.09; 203.12)	48.24 (−127.67; 224.16)	8.21 (−254.98; 271.41)	7.52 (−138.56; 153.60)	**Compressed air**	−0.91 (−127.45; 125.62)
56.10 (−16.96; 129.16)	47.33 (−74.88; 169.54)	7.30 (−223.48; 238.08)	6.61 (−66.39; 79.61)	−0.91 (−127.45; 125.62)	**Control group**

d) Health-related Quality of Life								
**Acupressure/ Reflexology**		0.07 (−1.75; 1.89)						**1.55 (0.22; 2.88)**
0.48 (−1.81; 2.78)	**Airflow**							1.07 (−0.80; 2.93)
0.83 (−0.89; 2.54)	0.34 (−2.19; 2.88)	**Lay foot manipulation**						−0.04 (−1.86; 1.79)
1.24 (−0.63; 3.12)	0.76 (−1.52; 3.04)	0.42 (−1.75; 2.58)	**Activity rehabilitation ​+ ​Behavioral psychoeducational ​+ ​integrative medicine**					0.31 (−1.01; 1.62)
1.28 (−0.60; 3.16)	0.80 (−1.49; 3.09)	0.45 (−1.72; 2.62)	0.04 (−1.83; 1.91)	**Activity rehabilitation ​+ ​Behavioral psychoeducational**				0.27 (−1.06; 1.60)
1.33 (−0.32; 2.99)	0.85 (−1.26; 2.96)	0.51 (−1.47; 2.48)	0.09 (−1.55; 1.73)	0.05 (−1.60; 1.70)	**Activity rehabilitation**			0.22 (−0.76; 1.20)
1.38 (−0.90; 3.65)	0.89 (−1.73; 3.51)	0.55 (−1.97; 3.07)	0.13 (−2.13; 2.40)	0.09 (−2.18; 2.37)	0.04 (−2.05; 2.13)	**Cannabidiol**		0.17 (−1.67; 2.02)
1.41 (−0.47; 3.28)	0.92 (−1.36; 3.21)	0.58 (−1.58; 2.75)	0.17 (−1.70; 2.03)	0.13 (−1.75; 2.00)	0.07 (−1.57; 1.72)	0.03 (−2.24; 2.30)	**Behavioral psychoeducational**	0.14 (−1.18; 1.46)
**1.55 (0.22;2.88)**	1.07 (−0.80; 2.93)	0.72 (−0.99; 2.44)	0.31 (−1.01; 1.62)	0.27 (−1.06; 1.60)	0.22 (−0.76; 1.20)	0.17 (−1.67; 2.02)	0.14 (−1.18; 1.46)	**Control group**

Activity rehabilitation ​+ ​Behavioral psychoeducational ​= ​AR ​+ ​BP; Activity rehabilitation ​+ ​Behavioral psychoeducational ​+ ​Integrative medicine ​= ​AR ​+ ​BP ​+ ​IM; Behavioral psychoeducational ​+ ​Integrative medicine ​= ​BP ​+ ​IM.

**Table 2:** P-score ranking probabilities of different interventions for 4 outcomes.

Interventions	Dyspnea severity		Anxiety		Exercise capacity		Health-related quality of life	
	P-score	Rank	P-score	Rank	P-score	Rank	P-score	Rank
HFNC	0.963	1	–	–	–	–	–	–
Acupressure or reflexology	0.837	2	0.261	8	0.653	2	0.880	1
Airflow	0.770	3	0.303	7	–	–	0.703	2
Opioids	0.636	4	–	–	0.414	4	–	–
Activity rehabilitation	0.600	5	0.963	1	0.748	1	0.404	6
Bilevel ventilation	0.570	6	–	–	–	–	–	–
Lay foot manipulation	0.533	7	0.529	4	–	–	0.591	3
Activity rehabilitation ​+ ​Behavioral psychoeducational	0.524	8	0.427	5	0.456	3	0.429	5
Activity rehabilitation ​+ ​Behavioral psychoeducational ​+ ​integrative medicine	0.514	9	0.606	3	–	–	0.445	4
Behavioral psychoeducational	0.461	10	–	–	–	–	0.375	8
Corticosteroids	0.424	11	–	–	–	–	–	–
Cannabidiol	0.394	12	–	–	–	–	0.399	7
Anxiolytics	0.333	13	0.207	9	–	–	–	–
Control group	0.329	14	0.422	6	0.340	6	0.274	9
Compressed air	0.226	15	–	–	0.389	5	–	–
Behavioral psychoeducational ​+ ​integrative medicine	0.198	16	0.782	2	–	–	–	–
Acupuncture	0.190	17	–	–	–	–	–	–

HFNC, high-flow nasal cannula.

HFNC (SMD ​= ​−1.91; 95% CI: −3.32 to −0.49; P-score ​= ​0.963; QOE: moderate) was significantly more effective than the control group in alleviating dyspnea severity, followed by Acupressure/Reflexology (SMD ​= ​−1.04; 95% CI: −2.02 to −0.06; P-score ​= ​0.837; QOE: very low).

However, there was considerable heterogeneity (τ^2^ ​= ​0.4243; I^2^ ​= ​83.9%; 95% CI: 77.7% to 88.3%) and measurable global inconsistency on a design-by-treatment model with random effects (Q statistic ​= ​14.48; *P* ​= ​0.0001). Two contrasts of local inconsistency were found, which indicates disagreement between indirect and direct evidence for Acupressure/Reflexology vs. Lay foot manipulation and Lay foot manipulation vs. the control group (*P* ​= ​0.002).

b) Anxiety

Only Activity rehabilitation (SMD ​= ​−0.64; 95% CI: −0.97 to −0.32; P-score ​= ​0.963; QOE: very low) was significantly more effective than the control group.

No heterogeneity was found (τ^2^ ​= ​0.0082; I^2^ ​= ​15.4%; 95% CI: 0.0% to 82.4%), and there was no measurable global or local inconsistency (Q statistic ​= ​0; *P* undefined).

c) Exercise capacity

No interventions were significantly more effective than the control group (QOE: very low). There was substantial heterogeneity (τ^2^ ​= ​3784.45; I^2^ ​= ​70.5%; 95% CI: 35.6% to 86.5%). No global or local inconsistency was found within the network (Q statistic ​= ​0; *P* undefined).

d) Health-related quality of life

Acupressure/Reflexology (SMD ​= ​1.55; 95% CI: 0.22 to 2.88; P-score ​= ​0.880; QOE: moderate) was the only intervention significantly more effective than the control group.

The analysis revealed considerable heterogeneity (τ^2^ ​= ​0.8357; I^2^ ​= ​89.6%; 95% CI: 81.9% to 94.0%) and global inconsistency (Q statistic ​= ​30.56; *P* ​< ​0.0001). Two contrasts of local inconsistency were also identified for Acupressure/Reflexology vs. Lay foot manipulation and Lay foot manipulation vs. the control group (*P* ​= ​0.0226).

### Publication bias

Funnel plots (Appendix 8) were used to assess publication bias for the outcomes of dyspnea severity, anxiety, exercise capacity, and HRQoL. Both the visual inspection of these plots and Egger's linear regression test indicated no significant evidence of publication bias, as confirmed by *P*-values greater than 0.05 across all outcomes.

### Sensitivity analysis

Sensitivity analyses were conducted by excluding studies with high ROB in the overall domain across all four outcomes: dyspnea severity, anxiety, exercise capacity, and HRQoL. The estimates (SMD) were similar to the base case analysis of dyspnea severity and anxiety with the control group, showing that the effect of RCTs with high ROB was not significant.

However, the original comparisons of the Acupressure or Reflexology with the control group substantially changed the overall estimates of exercise capacity and HRQoL, showing that the effect of RCTs with high ROB was significant. Results of Acupressure or Reflexology changed from MD ​= ​47.33; 95% CI: −74.88 to 169.54 into MD ​= ​47.33; 95% CI: 27.40 to 67.26 on exercise capacity, and from SMD ​= ​1.55; 95% CI: 0.22 to 2.88 into SMD ​= ​1.63; 95% CI: −0.77 to 4.02 on HRQoL while other interventions remained the overall estimates of exercise capacity and HRQoL significantly (Appendix 9).

### Risk of bias (ROB) assessment

The risk of bias judgments for each RCT is presented in Appendix 10. Overall, 20 trials were rated some concerns ROB, and 12 RCTs were judged as a high ROB. In addition, 10 studies were rated as a low ROB across all domains.

## Discussion

This network meta-analysis was conducted on 42 RCTs, including a total of 3832 patients, to examine the effectiveness of different treatments for dyspnea in advanced cancer. HFNC and Acupressure/Reflexology demonstrated statistically significant effectiveness compared to the control group on dyspnea relief. However, the quality of the evidence in the GRADE assessment was moderate for HFNC and very low for Acupressure/Reflexology. When comparing interventions, HFNC outperformed Activity rehabilitation ​+ ​Behavioral psychoeducational ​+ ​Integrative medicine, Corticosteroids, Compressed air, and Acupuncture, though GRADE assessments ranged from very low to low. Acupressure/Reflexology was significantly more effective than Compressed Air, albeit with very low-quality evidence. In terms of anxiety reduction, Activity rehabilitation showed a statistically significant improvement over the control group and all other interventions except for Behavioral psychoeducational ​+ ​Integrative medicine, even though all comparisons rated very low quality of evidence in the GRADE assessment. For exercise capacity, there was a slight but non-significant trend towards a beneficial effect compared to the control group and other interventions, all with very low evidence quality. Lastly, Acupressure/Reflexology was the only intervention that significantly enhanced health-related quality of life more than the control group, with a moderate quality by GRADE assessment.

Our study revealed several notable discrepancies when compared to previous research.^6^^,^^73^ Fan therapy (Airflow), which has been shown to significantly reduce dyspnea, did not yield statistically significant results in our analysis, likely due to the methodological differences used in each study. Previous study employed pairwise meta-analysis, which focus on direct comparisons between two interventions.^6^^,^^9^^,^^73^ Whereas our study utilized network meta-analysis (NMA) to simultaneously compare and rank multiple interventions, integrating both direct and indirect evidence. Similarly, our findings on Bilevel ventilation (BiPAP) diverged from prior research,^9^ which reported greater effectiveness compared to standard oxygen therapy. This variation may also stem from the type of data analyzed. As previous studies often relied on change-from-baseline data, while our study used post-intervention data, which may yield different interpretations of intervention effectiveness.^9^

Regarding opioids, our results contrast with studies that found them to be more effective in reducing dyspnea.^5^^,^^18^ This discrepancy could arise from variations in populations, as previous studies included broader cohorts, while ours focused specifically on advanced-stage cancer patients. Additionally, inconsistencies across studies that targeted exertional dyspnea versus dyspnea at rest may explain the differing outcomes. Furthermore, opioids may not have shown a significant effect in our study due to the issue of tolerance. Many advanced cancer patients are not opioid-naive and may have developed tolerance due to prior opioid use for pain management.^74^^,^^75^ These factors could explain why opioids ranked fourth in effectiveness in our analysis, showing no significant advantage over placebo or other pharmacological interventions.

For anxiety, we found that Activity rehabilitation significantly reduced anxiety, contrasting with previous research.^9^^,^^76^^,^^77^ This difference may be due to variations in the duration, intensity, and types of physical activities implemented across studies. Finally, while our study demonstrated that acupressure/reflexology significantly improved HRQoL, prior research reported negative effects of integrative medicine on HRQoL,^9^ likely attributable to differences in statistical methodologies.

The effectiveness of HFNC can be explained by its capacity to deliver humidified and heated gas at high flow rates, which decreases the work of breathing and enhances patient comfort.^78^ On the other hand, the success of Acupressure/Reflexology likely stems from its role in reducing stress and improving circulation, which can alleviate the physiological symptoms associated with dyspnea.^79^^,^^80^

Pharmacological interventions, such as opioids and corticosteroids, were less effective in our analysis, which are consistent with some existing literature.^10^^,^^81^ The multifactorial nature of dyspnea, especially in cancer patients, means that addressing the physical symptoms alone may not be enough to provide relief.^75^ This complexity could explain why opioids, despite being a first-line treatment for dyspnea,^70^ did not show statistically significant benefits in our study. Opioid tolerance, common in advanced cancer patients due to long-term pain management, might also play a role in diminishing their effectiveness for dyspnea relief.

In terms of anxiety management, effectiveness of Activity rehabilitation is well-documented. Physical activity promotes the release of endorphins and other neurochemicals that improve mood and reduce anxiety.^82^ This effect, combined with social interaction and support often included in rehabilitation programs, helps to alleviate feelings of isolation, which can exacerbate anxiety.^83^

### Implications for nursing practice and research

The findings from this study have important implications for clinical practice and future research in dyspnea management for advanced cancer patients. Given the significant benefits observed with HFNC and Acupressure/Reflexology, these interventions should be considered as part of an integrated approach to dyspnea management in this patient population. However, the variability in evidence quality underscores the need for further well-designed RCTs to confirm these results and to refine intervention protocols. Future research should focus on optimizing the combination of pharmacological and non-pharmacological treatments, tailoring interventions to individual patient needs, and exploring the long-term effects of these therapies on patient outcomes, particularly in diverse clinical settings.

### Limitations

This study has several strengths and limitations. To our best knowledge, this is the first network meta-analysis to compare the effectiveness of various interventions for dyspnea management in advanced cancer patients. Moreover, our inclusion of only RCTs further strengthens the validity and credibility of our findings.

However, the study has some limitations. Firstly, the quality of evidence for many interventions were rated as low or very low, which may have affected confidence in the findings. Secondly, the significant heterogeneity in patient populations, intervention protocols, and follow-up durations have complicated result interpretation and have contributed to variability in treatment effects. Lastly, the limited number of RCTs for specific interventions and small sample sizes have decreased the robustness of our conclusions.

## Conclusions

High-Flow Nasal Cannula (HFNC) significantly reduced dyspnea severity, followed by Acupressure/Reflexology. Acupressure/Reflexology also improving health-related quality of life. Additionally, Activity rehabilitation was effective in reducing anxiety. However, the overall low quality of evidence for many interventions limited confidence in these findings. Further high-quality trials are needed to confirm these results, strengthen the evidence base, and explore the potential benefits of combining different interventions for more comprehensive dyspnea management.

## CRediT authorship contribution statement

**An Thuy Vo**: Conceptualization, Methodology, Data curation, Formal analysis, Visualization, Investigation, Writing – Original draft preparation. **Kim-Ngan Thi Ta**: Conceptualization, Methodology, Software, Writing – Reviewing and Editing. **Kai-Jen Chuang**: Data curation, Supervision, Software, Writing – Reviewing and Editing. All authors had full access to all the data in the study, and the corresponding author had final responsibility for the decision to submit for publication. The corresponding author attests that all listed authors meet authorship criteria and that no others meeting the criteria have been omitted.

## Ethics statement

Not required.

## Funding

This study was supported by the National Science and Technology Council (Grant No. 113-2918-I-038-004). The funders had no role in considering the study design or in the collection, analysis, interpretation of data, writing of the report, or decision to submit the article for publication.

## Data availability statement

Data availability is not applicable to this article as no new data were created or analyzed in this study.

## Declaration of generative AI and AI-assisted technologies in the writing process

No AI tools/services were used during the preparation of this work.

## Declaration of competing interest

The authors declare no conflict of interest.

## Footnotes

Appendix A Supplementary data to this article can be found online at https://doi.org/10.1016/j.apjon.2025.100671.
