# Supplementary material for: Comparative effectiveness of pharmacological and non-pharmacological interventions for dyspnea management in advanced cancer: A systematic review and network meta-analysis
Source: Asia Pac J Oncol Nurs. 2025 Feb 18;12:100671. doi: 10.1016/j.apjon.2025.100671 (PMC11931311; doi:10.1016/j.apjon.2025.100671)
Supplement: Multimedia component 1 [file mmc1.docx]

**Supplemental Online Content**

[Appendix 1. Search strategy 2](#_Toc190356371)

[Appendix 2. Characteristics of the included studies 8](#_Toc190356372)

[Appendix 3. Network graphs of intervention comparisons for 4 outcomes: dyspnea severity, anxiety, exercise capacity, health-related quality of life 18](#_Toc190356373)

[Appendix 4. Global Inconsistency of all outcomes 20](#_Toc190356374)

[Appendix 5. Local inconsistency in network 21](#_Toc190356375)

[Appendix 6. Certainty of direct evidence assessment 27](#_Toc190356376)

[Appendix 7. Certainty of network evidence assessment 31](#_Toc190356377)

[Appendix 8. Funnel plots for all outcomes 46](#_Toc190356378)

[Appendix 9. Sensitivity analysis 48](#_Toc190356379)

[Appendix 10. Risk of bias of each RCT 51](#_Toc190356380)

# Appendix 1. Search strategy

**Keywords for randomized controlled trials since the date last searched of the previous network meta-analyses** ^9,10^ **(May 1st, 2020)**

| **Databases** | **Search queries** | **Results** |
| --- | --- | --- |
| **PubMed** | (dyspnea + malignancy) AND (("2020/05/01"(Date - Publication): "3000"(Date - Publication))) AND (randomizedcontrolledtrial(Filter)) | 73 |
|  | ((cancer(Title/Abstract) OR carcinoma(Title/Abstract) OR malignancy(Title/Abstract) OR neoplasm(Title/Abstract) OR tumor(Title/Abstract) OR oncology(Title/Abstract) OR metastasis(Title/Abstract) OR "end-stage cancer"(Title/Abstract) OR "late-stage"(Title/Abstract) OR "Advanced cancer"(Title/Abstract) OR "terminal stages"(Title/Abstract)) AND ("Palliative interventions"(Title/Abstract) OR "Adrenergic beta-Agonists"(Title/Abstract) OR "Bronchodilator Agents"(Title/Abstract) OR albuterol(Title/Abstract) OR arformoterol(Title/Abstract) OR aclidinium(Title/Abstract) OR "aerosolized medications"(Title/Abstract) OR alprazolam(Title/Abstract) OR amiloride(Title/Abstract) OR aminophylline(Title/Abstract) OR Analgesics(Title/Abstract) OR Anticonvulsants(Title/Abstract) OR "Anti-Inflammatory Agents"(Title/Abstract) OR Antimuscarinics(Title/Abstract) OR "Antipsychotic Agents"(Title/Abstract) OR arformoterol(Title/Abstract) OR aripiprazole(Title/Abstract) OR asenapine(Title/Abstract) OR atropine(Title/Abstract) OR beclomethasone(Title/Abstract) OR brexpiprazole(Title/Abstract) OR Bronchodilator(Title/Abstract) OR budesonide(Title/Abstract) OR bumetanide(Title/Abstract) OR bupropion(Title/Abstract) OR Buspirone(Title/Abstract) OR caffeine(Title/Abstract) OR cariprazine(Title/Abstract) OR celecoxib(Title/Abstract) OR chlorpromazine(Title/Abstract) OR ciclesonide(Title/Abstract) OR citalopram(Title/Abstract) OR clozapine(Title/Abstract) OR desvenlafaxine(Title/Abstract) OR dexamethasone(Title/Abstract) OR diazepam(Title/Abstract) OR diclofenac(Title/Abstract) OR diflusinal(Title/Abstract) OR Diuretics(Title/Abstract) OR duloxetine(Title/Abstract) OR escitalopram(Title/Abstract) OR "ethacrynic acid"(Title/Abstract) OR etodolac(Title/Abstract) OR fenoprofen(Title/Abstract) OR flunisolide(Title/Abstract) OR fluoxetine(Title/Abstract) OR flurbiprofen(Title/Abstract) OR fluticasone(Title/Abstract) OR fluvoxamine(Title/Abstract) OR formoterol(Title/Abstract) OR gabapentin(Title/Abstract) OR glycopyrrolate(Title/Abstract) OR haloperidol(Title/Abstract) OR hydrochlorothiazide(Title/Abstract) OR hydrocodone(Title/Abstract) OR hydrocortisone(Title/Abstract) OR ibuprofen(Title/Abstract) OR iloperidone(Title/Abstract) OR indapamide(Title/Abstract) OR indomethacin(Title/Abstract) OR Inhalators(Title/Abstract) OR "inhaled medications"(Title/Abstract) OR Inhaler(Title/Abstract) OR ipratropium(Title/Abstract) OR ketoprofen(Title/Abstract) OR ketorolac(Title/Abstract) OR levalbuterol(Title/Abstract) OR levomilnacipran(Title/Abstract) OR Lidocaine(Title/Abstract) OR lorazepam(Title/Abstract) OR lurasidone(Title/Abstract) OR meloxicam(Title/Abstract) OR methylprednisolone(Title/Abstract) OR Methylxanthines(Title/Abstract) OR metolazone(Title/Abstract) OR midazolam(Title/Abstract) OR milnacipran(Title/Abstract) OR mirtazapine(Title/Abstract) OR mometasone(Title/Abstract) OR morphine(Title/Abstract) OR nabumetone(Title/Abstract) OR naproxen(Title/Abstract) OR "nebulized medications"(Title/Abstract) OR nebulizer(Title/Abstract) OR Non-Steroidal(Title/Abstract) OR olanzapine(Title/Abstract) OR olodaterol(Title/Abstract) OR oxaprozin(Title/Abstract) OR oxymorphone(Title/Abstract) OR paliperidone(Title/Abstract) OR paroxetine(Title/Abstract) OR Phenylpropionates(Title/Abstract) OR pimavanserin(Title/Abstract) OR piroxicam(Title/Abstract) OR prednisone(Title/Abstract) OR Pregabalin(Title/Abstract) OR prochlorperazine(Title/Abstract) OR promethazine(Title/Abstract) OR Propionates(Title/Abstract) OR quetiapine(Title/Abstract) OR risperidone(Title/Abstract) OR salsalate(Title/Abstract) OR scopolamine(Title/Abstract) OR sertraline(Title/Abstract) OR spironolactone(Title/Abstract) OR Steroids(Title/Abstract) OR sulindac(Title/Abstract) OR tapentadol(Title/Abstract) OR terbutaline(Title/Abstract) OR theophylline(Title/Abstract) OR thioridazine(Title/Abstract) OR tiotropium(Title/Abstract) OR tolmetin(Title/Abstract) OR torsemide(Title/Abstract) OR triamterene(Title/Abstract) OR umeclidinium(Title/Abstract) OR Vaporizers(Title/Abstract) OR venlafaxine(Title/Abstract) OR vilanterol(Title/Abstract) OR ziprasidone(Title/Abstract) OR atrovent(Title/Abstract) OR benzodiazepine*(Title/Abstract) OR buprenorphine(Title/Abstract) OR chlorpromazine(Title/Abstract) OR codeine(Title/Abstract) OR corticosteroids(Title/Abstract) OR dextromoramide(Title/Abstract) OR dextropropoxyphene(Title/Abstract) OR diamorphine(Title/Abstract) OR dihydrocodeine(Title/Abstract) OR dipipanone(Title/Abstract) OR "drug mechanism"(Title/Abstract) OR "drug therapy"(Title/Abstract) OR fentanyl(Title/Abstract) OR furosemide(Title/Abstract) OR hydromorphone(Title/Abstract) OR meptazinol(Title/Abstract) OR methadone(Title/Abstract) OR nalbuphine(Title/Abstract) OR opiate*(Title/Abstract) OR opioid*(Title/Abstract) OR OTFC(Title/Abstract) OR oxycodone(Title/Abstract) OR papaveretum(Title/Abstract) OR pentazocine(Title/Abstract) OR pethidine(Title/Abstract) OR "pharmacologic actions"(Title/Abstract) OR Pharmacologic*(Title/Abstract) OR "phenothiazine derivative"(Title/Abstract) OR phenothiazines(Title/Abstract) OR puffer*(Title/Abstract) OR remifentanil(Title/Abstract) OR steroids(Title/Abstract) OR sufentanil(Title/Abstract) OR tramadol(Title/Abstract) OR "pharmacological treatment"(Title/Abstract) OR "drug therapy"(Title/Abstract) OR alfentanil(Title/Abstract)) AND (dyspnea*(Title/Abstract) OR breathlessness(Title/Abstract) OR "shortness of breath"(Title/Abstract) OR "respiratory distress"(Title/Abstract))) AND (("2020/05/01"(Date - Publication) : "3000"(Date - Publication))) Filters: Randomized Controlled Trial | 12 |
|  | ((cancer(Title/Abstract) OR carcinoma(Title/Abstract) OR malignancy(Title/Abstract) OR neoplasm(Title/Abstract) OR tumor(Title/Abstract) OR oncology(Title/Abstract) OR metastasis(Title/Abstract) OR "end-stage cancer"(Title/Abstract) OR "late-stage"(Title/Abstract) OR "Advanced cancer"(Title/Abstract) OR "terminal stages"(Title/Abstract)) AND (Palliative interventions(Title/Abstract) OR nonpharmacological*(Title/Abstract) OR Complementary Therapies(Title/Abstract) OR "fan therapy"(Title/Abstract) OR oxygen(Title/Abstract) OR acupuncture(Title/Abstract) OR nursing(Title/Abstract) OR meditation(Title/Abstract) OR mindful*(Title/Abstract) OR rehab*(Title/Abstract) OR "music therapy"(Title/Abstract) OR yoga(Title/Abstract) OR respiratory(Title/Abstract) OR "Behavioral Therapy"(Title/Abstract) OR "Cognitive behavioral therapy (CBT)"(Title/Abstract) OR "Non-invasive ventilation"(Title/Abstract) OR Exercise(Title/Abstract) OR "Physical activity"(Title/Abstract) OR acupressure(Title/Abstract) OR mindful*(Title/Abstract) OR rehabilitation(Title/Abstract) OR reiki(Title/Abstract) OR massage(Title/Abstract) OR "Compressed Air"(Title/Abstract) OR "room air"(Title/Abstract) OR "room environment"(Title/Abstract) OR "water spray"(Title/Abstract) OR helium(Title/Abstract) OR heliox(Title/Abstract) OR "airway pressure"(Title/Abstract) OR "Oxygen Inhalation Therapy"(Title/Abstract) OR oxygen(Title/Abstract) OR "respiratory therapy"(Title/Abstract) OR ventilation(Title/Abstract) OR "pressure respiration"(Title/Abstract) OR "high flow"(Title/Abstract) OR Bipap(Title/Abstract) OR cpap(Title/Abstract) OR "Cognitive Behavioral Therapy"(Title/Abstract) OR "Behavioral Therapy"(Title/Abstract) OR behavio*(Title/Abstract) OR "management strategies"(Title/Abstract) OR nurse(Title/Abstract) OR nursing(Title/Abstract) OR multidisciplinary(Title/Abstract) OR clinic(Title/Abstract) OR psychosocial(Title/Abstract) OR psychoeducational(Title/Abstract) OR Psychotherapy(Title/Abstract) OR biofeedback(Title/Abstract) OR "adaptation strategies"(Title/Abstract) OR "energy conservation"(Title/Abstract) OR "activity pacing"(Title/Abstract) OR "teaching coping"(Title/Abstract) OR "Relaxation Therapy"(Title/Abstract) OR relaxation(Title/Abstract) OR "distraction therapy"(Title/Abstract) OR Exercise(Title/Abstract) OR "Exercise Movement Techniques"(Title/Abstract) OR "breathing techniques"(Title/Abstract) OR "breathing exercise"(Title/Abstract) OR "Tai Chi"(Title/Abstract)) AND (dyspnea*(Title/Abstract) OR breathlessness(Title/Abstract) OR "shortness of breath"(Title/Abstract) OR "respiratory distress"(Title/Abstract))) AND (("2020/05/01"(Date - Publication) : "3000"(Date - Publication))) Filters: Randomized Controlled Trial | 44 |
| **Cochrane Library: Cochrane Central Register of Controlled Trials (CENTRAL)** | (breathlessness OR dyspnea OR shortness of breath) AND advanced cancer in Title Abstract Keyword - with Publication Year from 2020 to 2024, with Cochrane Library publication date Between May 2020 and Jan 2024, in Trials (Word variations have been searched) | 219 |
|  | (cancer OR carcinoma OR malignancy OR neoplasm OR tumor OR oncology OR metastasis OR "end-stage cancer" OR "late-stage" OR "Advanced cancer" OR "terminal stages") AND ("Palliative interventions" OR "Adrenergic beta-Agonists" OR "Bronchodilator Agents" OR albuterol OR arformoterol OR aclidinium OR "aerosolized medications" OR alprazolam OR amiloride OR aminophylline OR Analgesics OR Anticonvulsants OR "Anti-Inflammatory Agents" OR Antimuscarinics OR "Antipsychotic Agents" OR arformoterol OR aripiprazole OR asenapine OR atropine OR beclomethasone OR brexpiprazole OR Bronchodilator OR budesonide OR bumetanide OR bupropion OR Buspirone OR caffeine OR cariprazine OR celecoxib OR chlorpromazine OR ciclesonide OR citalopram OR clozapine OR desvenlafaxine OR dexamethasone OR diazepam OR diclofenac OR diflusinal OR Diuretics OR duloxetine OR escitalopram OR "ethacrynic acid" OR etodolac OR fenoprofen OR flunisolide OR fluoxetine OR flurbiprofen OR fluticasone OR fluvoxamine OR formoterol OR gabapentin OR glycopyrrolate OR haloperidol OR hydrochlorothiazide OR hydrocodone OR hydrocortisone OR ibuprofen OR iloperidone OR indapamide OR indomethacin OR Inhalators OR "inhaled medications" OR Inhaler OR ipratropium OR ketoprofen OR ketorolac OR levalbuterol OR levomilnacipran OR Lidocaine OR lorazepam OR lurasidone OR meloxicam OR methylprednisolone OR Methylxanthines OR metolazone OR midazolam OR milnacipran OR mirtazapine OR mometasone OR morphine OR nabumetone OR naproxen OR "nebulized medications" OR nebulizer OR Non-Steroidal OR olanzapine OR olodaterol OR oxaprozin OR oxymorphone OR paliperidone OR paroxetine OR Phenylpropionates OR pimavanserin OR piroxicam OR prednisone OR Pregabalin OR prochlorperazine OR promethazine OR Propionates OR quetiapine OR risperidone OR salsalate OR scopolamine OR sertraline OR spironolactone OR Steroids OR sulindac OR tapentadol OR terbutaline OR theophylline OR thioridazine OR tiotropium OR tolmetin OR torsemide OR triamterene OR umeclidinium OR Vaporizers OR venlafaxine OR vilanterol OR ziprasidone OR atrovent OR benzodiazepine* OR buprenorphine OR chlorpromazine OR codeine OR corticosteroids OR dextromoramide OR dextropropoxyphene OR diamorphine OR dihydrocodeine OR dipipanone OR "drug mechanism" OR "drug therapy" OR fentanyl OR furosemide OR hydromorphone OR meptazinol OR methadone OR nalbuphine OR opiate* OR opioid* OR OTFC OR oxycodone OR papaveretum OR pentazocine OR pethidine OR "pharmacologic actions" OR Pharmacologic* OR "phenothiazine derivative" OR phenothiazines OR puffer* OR remifentanil OR steroids OR sufentanil OR tramadol OR "pharmacological treatment" OR "drug therapy" OR alfentanil) AND (dyspnea* OR breathlessness OR "shortness of breath" OR "respiratory distress") in Title Abstract Keyword - with Publication Year from 2020 to 2024, with Cochrane Library publication date Between May 2020 and Mar 2024, in Trials (Word variations have been searched) | 554 |
|  | (cancer OR carcinoma OR malignancy OR neoplasm OR tumor OR oncology OR metastasis OR "end-stage cancer" OR "late-stage" OR "Advanced cancer" OR "terminal stages") AND (Palliative interventions OR nonpharmacological* OR Complementary Therapies OR "fan therapy" OR oxygen OR acupuncture OR nursing OR meditation OR mindful* OR rehab* OR "music therapy" OR yoga OR respiratory OR "Behavioral Therapy" OR "Cognitive behavioral therapy (CBT)" OR "Non-invasive ventilation" OR Exercise OR "Physical activity" OR acupressure OR mindful* OR rehabilitation OR reiki OR massage OR "Compressed Air" OR "room air" OR "room environment" OR "water spray" OR helium OR heliox OR "airway pressure" OR "Oxygen Inhalation Therapy" OR oxygen OR "respiratory therapy" OR ventilation OR "pressure respiration" OR "high flow" OR Bipap OR cpap OR "Cognitive Behavioral Therapy" OR "Behavioral Therapy" OR behavio* OR "management strategies" OR nurse OR nursing OR multidisciplinary OR clinic OR psychosocial OR psychoeducational OR Psychotherapy OR biofeedback OR "adaptation strategies" OR "energy conservation" OR "activity pacing" OR "teaching coping" OR "Relaxation Therapy" OR relaxation OR "distraction therapy" OR Exercise OR "Exercise Movement Techniques" OR "breathing techniques" OR "breathing exercise" OR "Tai Chi") AND (dyspnea* OR breathlessness OR "shortness of breath" OR "respiratory distress") in Title Abstract Keyword - with Publication Year from 2020 to 2024, with Cochrane Library publication date Between May 2020 and Mar 2024, in Trials (Word variations have been searched) | 994 |
| **Hinari via Research4Life** | ((TitleCombined:(\(cancer OR carcinoma OR malignancy OR neoplasm OR tumor OR oncology\)) OR (Abstract:(\(cancer OR carcinoma OR malignancy OR neoplasm OR tumor OR oncology\))) AND ((TitleCombined:(\(breathlessness OR dyspnea OR shortness OR of OR breath OR respiratory OR distress\)) OR (Abstract:(\(breathlessness OR dyspnea OR shortness of breath OR respiratory distress\))) AND ((TitleCombined:(randomized controlled trial)) OR (Abstract:(randomized controlled trial))) | 256 |
| **ResearchGate** | (breathlessness OR dyspnea OR shortness of breath) AND advanced cancer AND Randomized Controlled Trials AND intervention | 1000 |

# Appendix 2. Characteristics of the included studies

| **No** | **Authors, year** | **Study design** | **Patient Characteristics Age (mean ± SD); Gender, n (%)** | **Sample size** | **Cancer Types** | **Outcomes** | | | | **Intervention Group** | **Control Group** | **Duration follow-up** |
| --- | --- | --- | --- | --- | --- | --- | --- | --- | --- | --- | --- | --- |
|  |  |  |  |  |  | **Dyspnea Severity** | **Anxiety** | **Exercise Capacity** | **HRQOL** |  |  |  |
| **Airflow** | | | | | | | | | | | | |
| 1 | Kako et al, 2018 | RCT | Age: 69 ± 10.21  Male: 22 (55%)  Female: 18 (45%) | 40 | Lung, colorectal, breast, stomach, esophagus, gallbladder/bile duct, pancreas, head and neck, prostate | x | x |  |  | Fan to face  (n = 20) | Fan to legs  (n = 20) | 5 minutes |
| 2 | Ting et al, 2020 | Crossover RCT | Age: 51 ± 18.26  Male: 22 (45.8%)  Female: 26 (54.2%) | 48 | Lung, gastrointestinal, genitourinary, head and neck, breast, lymphoma, melanoma, sarcoma | x |  |  |  | Fan to face  (n = 24) | Fan to legs  (n = 24) | 5 minutes |
| 3 | Kocatepe et al, 2021 | RCT | Age: 65 ± 8.65  Male: 83 (86.45%)  Female: 7 (13.5%) | 96 | Lung cancer | x |  |  | x | Fan to face  (n = 47) | Standard care (n = 49) | 14 days |
| **Acupuncture** | | | | | | | | | | | | |
| 4 | Vickers et al, 2005 | RCT | Age: 64.7 ± 12.2  Male: 17 (38%)  Female: 28 (62%) | 47 | Breast, lung | x |  |  |  | Acupuncture (n=25) | Sham acupuncture (n=22) | 1 week |
| **Acupressure or Reflexology** | | | | | | | | | | | | |
| 5 | Wyatt et al, 2012 | RCT | Age: 56 ± 10.85  Female: (100%) | 286 | Breast | x | x |  | x | Reflexology  (n = 95)  Lay Foot Manipulation  (n = 95) | Standard care  (n = 96) | 11 weeks |
| 6 | Dogan et al, 2020 | RCT | Age: 61.1 ± 8.04  Male: 53 (88%)  Female: 7 (12%) | 60 | Lung | x |  | x | x | Acupressure  (n = 29) | Standard care  (n = 31) | 4 weeks |
| **Activity rehabilitation + Behavioral psychoeducational** | | | | | | | | | | | | |
| 7 | Chan et al, 2011 | RCT | Age: NR  Male: 116 (83%)  Female: 24 (17%) | 140 | Lung | x | x |  |  | Respiratory training and Psychoeducational intervention  (n = 70) | Usual care  (n = 70) | 12 weeks |
| 8 | Dhillon et al, 2017 | RCT | Age: 60 ± 9.07  Male: 61 (55%)  Female: 50 (45%) | 111 | Lung | x | x | x | x | Physical activity programme plus General health education materials  (n=56) | Usual care, including General health education materials only  (n=55) | 6 months |
| 9 | Bade et al, 2021 | RCT | Age: 64.88 ± 8.69  Male: 10 (25%)  Female: 30 (75%) | 40 | Lung | x |  |  | x | A combined intervention, including education, physical activity, text messaging  (n = 20) | Usual care  (n = 20) | 12 weeks |
| **Activity rehabilitation + Behavioral psychoeducational + Integrative medicine** | | | | | | | | | | | | |
| 10 | Farquhar et al, 2014 | Crossover RCT | Age: 69 ± 11.5  Male: 26 (39%)  Female: 41 (61%) | 67 | Lung, mesothelioma, breast, GI, GU, lymphoma, others | x | x |  | x | Breathlessness Intervention Service  (n = 35) | Usual care  (n = 32) | 2 weeks |
| 11 | Yorke et al, 2015 | RCT | Age: 67.7 ± 9.6  Male: 47 (47%)  Female: 54 (53%) | 101 | Lung | x | x |  |  | Respiratory Distress Symptom Intervention  (n=50) | Usual care  (n = 51) | 12 weeks |
| 12 | Yates et al, 2020 | RCT | Age: 67.9 ± 9.6  Male: 91 (63.2%)  Female: 53 (36.8%) | 144 | Lung | x | x |  |  | Non-pharmacological Interventions  (n = 81) | Standard care  (n = 63) | 8 weeks |
| 13 | Narayanan et al, 2023 | RCT | Most patients aged between 41 and 60 years.  Male: 70 (78%)  Female: 20 (22%) | 90 | Oral, Laryngopharynx, Other Head and Neck cancer | x |  |  | x | video-based education  (n = 45) | verbal instruction  (n = 45) | 3 weeks |
| 14 | Yorke et al, 2023 | RCT | Age: 69 ± 9.47  Male: 131 (49.8%)  Female: 132 (50.2%) | 263 | Lung | x | x |  |  | Respiratory Distress Symptom Intervention  (n=132) | Usual care  (n = 131) | 12 weeks |
| **Behavioral psychoeducational** | | | | | | | | | | | | |
| 15 | Moore et al, 2002 | RCT | Age: 67 ± 8.8  Male: 140 (69.3%)  Female: 62 (30.7%) | 202 | Lung, mesothelioma | x |  |  | x | Nurse-led follow-up  (n = 99) | Conventional medical follow-up  (n = 103) | 3 months |
| 16 | Bordeleau et al, 2003 | RCT | Age: 50 ± 8.94  Female: (100%) | 215 | Breast | x |  |  | x | Psychosocial Support  (n = 145) | Usual care  (n=70) | 12 months |
| **Behavioral psychoeducational + Integrative medicine** | | | | | | | | | | | | |
| 17 | Mosher et al, 2019 | RCT | Age: 62.6 ± 12.23  Male: 14 (56%)  Female: 11 (44%) | 50 | Lung | x |  |  |  | Telephone-based Acceptance and Commitment Therapy  (n = 25) | Education/Support  (n = 25) | 6 weeks |
| **Bilevel ventilation** | | | | | | | | | | | | |
| 18 | Nava et al, 2013 | RCT | Age: 70.5 ± 11.51  Male: 124 (62%)  Female: 76 (38%) | 200 | Lung, Gastrointestinal, breast, head, and neck, other | x |  |  |  | Bilevel ventilation  (n = 99) | Standard supplemental oxygen  (n = 101) | 48 hours |
| **Compressed air** | | | | | | | | | | | | |
| 19 | Booth et al, 1996 | Crossover RCT | Age: 71.5 ± 8.42  Male: 22 (58%)  Female: 16 (42%) | 38 | Lung, mesothelioma, others | x |  |  |  | Compressed air  (n = 18) | Standard supplemental oxygen  (n = 20) | 15 minutes |
| 20 | Bruera et al, 2003 | Crossover RCT | Age: 62 ± 9.13  Male: 21 (64%)  Female: 12 (36%) | 33 | Lung, other | x |  | x |  | Compressed air  (n = 17) | Standard supplemental oxygen  (n = 16) | 11 minutes |
| 21 | Philip et al, 2006 | Crossover RCT | Age: 61.25 ± 10.88  Male: 31 (61%)  Female: 20 (39%) | 51 | Lung, breast, colon, others | x |  |  |  | Compressed air  (n = 27) | Standard supplemental oxygen  (n = 24) | 15 minutes |
| **Activity and Rehabilitation Interventions** | | | | | | | | | | | | |
| 22 | Hwang et al, 2012 | RCT | Age: 59.85 ± 7.22  Male: 12 (50%)  Female: 12 (50%) | 24 | Lung | x |  |  | x | Exercise training  (n = 13) | Usual care  (n = 11) | 8 weeks |
| 23 | Henke et al, 2013 | RCT | NR | 29 | Lung | x |  | x | x | Strength and endurance training, Breathing techniques  (n =18) | Respiratory training and exercise therapy  (n =11) | 9 weeks |
| 24 | Molassiotis et al, 2014 | RCT | Age: 69.5 ± 8.35  Male: 37 (80%)  Female: 9 (20%) | 47 | Lung, mesothelioma | x |  |  |  | Inspiratory muscle training  (n = 24) | Usual care  (n = 23) | 12 weeks |
| 25 | Jastrzębski et al, 2015 | RCT | Age: 59 ± 7  Male: 10 (83%)  Female: 2 (17%) | 20 | Lung | x |  | x | x | Physical rehabilitation  (n = 12) | No treatment  (n = 8) | 8 weeks |
| 26 | Nakano et al, 2019 | Crossover RCT | Age: 70 ± 6.3  Male: 17 (85%)  Female: 3 (15%) | 20 | Lung, gastrointestinal, genitourinary, head and neck, breast. Lymphoma | x |  |  |  | Transcutaneous electrical nerve stimulation  (n = 20) | Usual care  (n = 20) | 6 days |
| 27 | Rutkowska et al, 2019 | RCT | Age: 60 ± 7.5  Male: 37 (80%)  Female: 9 (20%) | 30 | Lung | x |  | x |  | Exercise therapy and respiratory training  (n =20) | Usual care  (n =10) | 6 weeks |
| 28 | Molassiotis et al, 2021 | RCT | Age: 56.84 ± 9.45  Male: 116 (74.4%)  Female: 40 (25.6%) | 156 | Lung | x | x |  | x | Qigong training  (n = 78) | Usual care  (n = 78) | 12 weeks |
| 29 | Rodriguez et al, 2021 | RCT | Age: 70.44 ± 9.29  Male: 62 (58.5%)  Female: 44 (41.5%) | 113 | Lung, Breast, Digestive system cancer | x | x |  |  | Comprehensive functional rehabilitation programme  (n = 61) | Only standard drug treatment  (n = 52) | 45 minutes |
| 30 | Xu et al, 2023 | RCT | NR | 205 | Lung | x |  | x |  | Strength and endurance training, Breathing techniques  (n =18) | Respiratory training and exercise therapy  (n =11) | 9 weeks |
| **High Flow Nasal Cannula** | | | | | | | | | | | | |
| 31 | Xu et al, 2021 | RCT | Age: 58.85 ± 9.93  Male: 33 (55%)  Female: 27 (45%) | 60 | NR | x |  |  |  | High Flow Nasal Cannula oxygen therapy  (n = 30) | Conventional nasal catheter oxygen therapy  (n = 30) | 72 hours |
| **Anxiolytics** | | | | | | | | | | | | |
| 32 | Peoples et al, 2015 | RCT | Age: 64 ± 9.85  Male: 184 (49%)  Female: 195 (51%) | 379 | Lung, Breast, Gastrointestinal, Other | x | x |  |  | Buspirone  (N=187) | Placebo  (N=192) | 28 days |
| **Cannabidiol** | | | | | | | | | | | | |
| 33 | Hardy et al, 2022 | RCT | Age: 64.6 ± 12.8  Male: 75 (52.8%)  Female: 67 (47.2%) | 142 | Prostate, Breast, Colorectal, Gynecologic, Lung, Hematologic, Others | x |  |  | x | Cannabidiol oil  (N = 58) | Placebo  (N = 63) | 28 days |
| **Corticosteroids** | | | | | | | | | | | | |
| 34 | Hui et al, 2016 (Corticosteroids) | RCT | Age: 63 ± 6.92  Male: 16 (39%)  Female: 25 (61%) | 41 | NSCLC, SCLC, Mesothelioma, Others | x |  |  |  | Dexamethasone  (n=20) | Placebo  (n=21) | 14 days |
| 35 | Hui et al, 2023 | RCT | Age: 65 ± 9.74  Male: 54 (42%)  Female: 74 (58%) | 128 | Breast, Gastrointestinal, Genitourinary, Respiratory, Other | x |  |  |  | Dexamethasone  (n=85) | Placebo  (n=43) | 14 days |
| **Opioids** | | | | | | | | | | | | |
| 36 | Bruera et al, 1993 | Crossover RCT | NR | 10 | Lung (others not specified) | x |  |  |  | Morphine (n=10) | Placebo  (n=10) | 60 minutes |
| 37 | Mazzocato et al, 1999 | Crossover RCT | Age: 73 ± 5.68  Male: 5 (56%)  Female: 4 (44%) | 9 | Lung, breast, bladder | x |  |  |  | S/C Morphine  (n=9) | Placebo  (n=9) | 45 minutes |
| 38 | Charles et al, 2008 | Crossover RCT | Age: 69 ± 9.36  Male: 11 (55%)  Female: 9 (45%) | 20 | Breast, Lung, Mesothelioma, Prostate, Renal Cancer | x |  |  |  | Nebulized hydromorphone  (n = 6)  Systemic hydromorphone  (n = 7) | Nebulized saline  (n = 7) | 60 minutes |
| 39 | Pinna et al, 2013 | Crossover RCT | Age: 65.2 ± 10.4  Male: 11 (84.6%)  Female: 2 (15.4%) | 13 | Breast, Kidney, Lung, Stomach | x |  | x |  | Oral transmucosal fentanyl citrate (OTFC)  (n=11) | Placebo  (n=11) | 7 Days |
| 40 | Hui et al, 2014 | RCT | Age: 55 ± 12.84  Male: 9 (45%)  Female: 11 (55%) | 20 | Breast, Gastrointestinal, Genitourinary, Gynecologic, Lung, Sarcoma | x |  | x |  | Fentany  (n=10) | Placebo  (n=10) | 160 minutes |
| 41 | Hui et al, 2016 | RCT | Age: 52.4 ± 2.54  Male: 11 (45.8%)  Female: 13 (54.2%) | 24 | Breast, Gastrointestinal, Genitourinary, Gynecologic, Lung, Hematologic, Others | x |  | x |  | Fentanyl Pectin Nasal Spray (FPNS)  (n=12) | Placebo  (n=12) | 172 minutes |
| 42 | Hui et al, 2017 | RCT | Age: 55 ± 10.97  Male: 8 (40%)  Female: 12 (60%) | 20 | Breast, Gastrointestinal, Genitourinary, Gynecologic, Lung, Others | x |  | x |  | Fentanyl Buccal Tablet (FBT)  (n = 9) | Placebo  (n=11) | 6 minutes |

NR: Not Reported

# Appendix 3. Network graphs of intervention comparisons for 4 outcomes: dyspnea severity, anxiety, exercise capacity, health-related quality of life

3a) Dyspnea severity


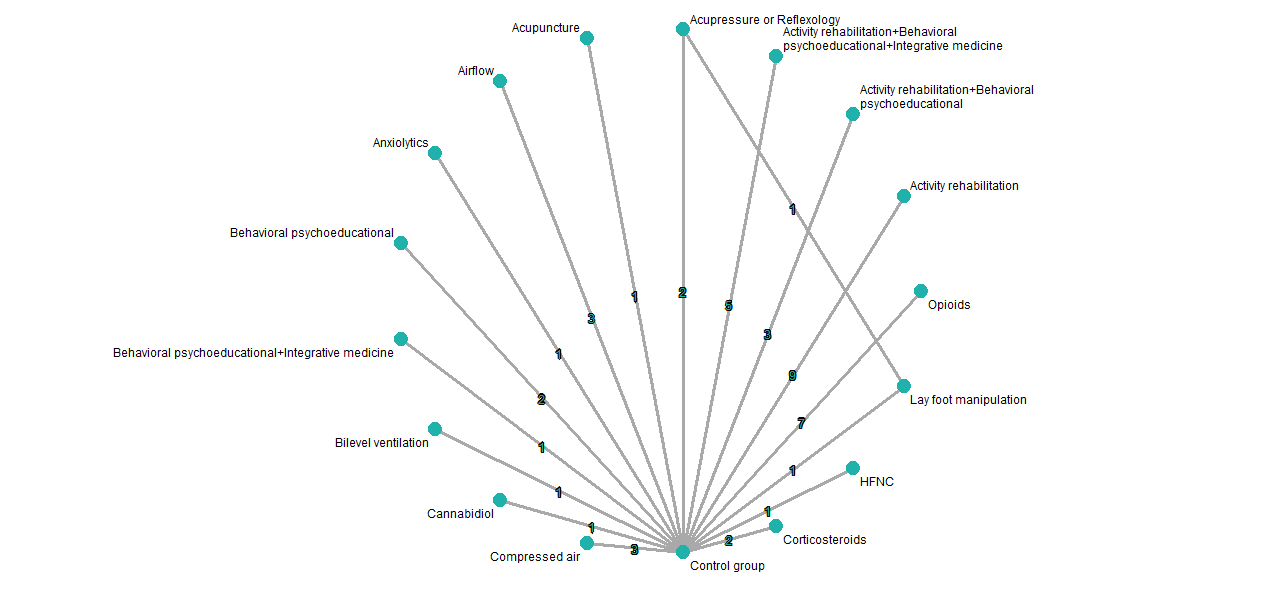


3b) Anxiety


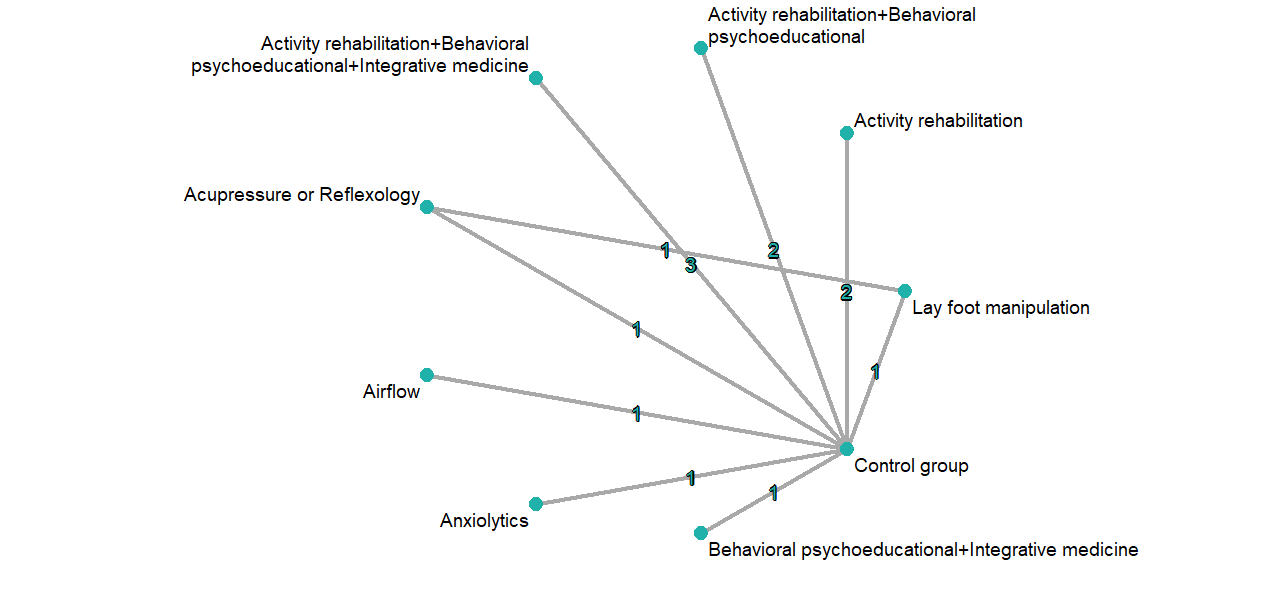


3c) Exercise capacity


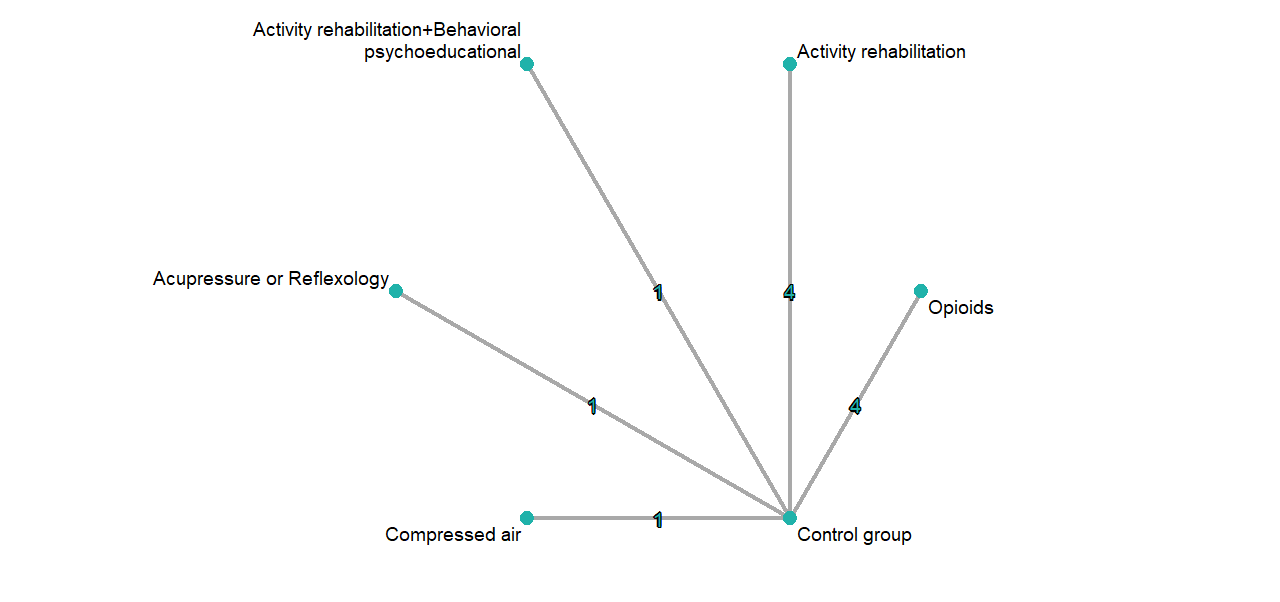


3d) Health-related quality of life


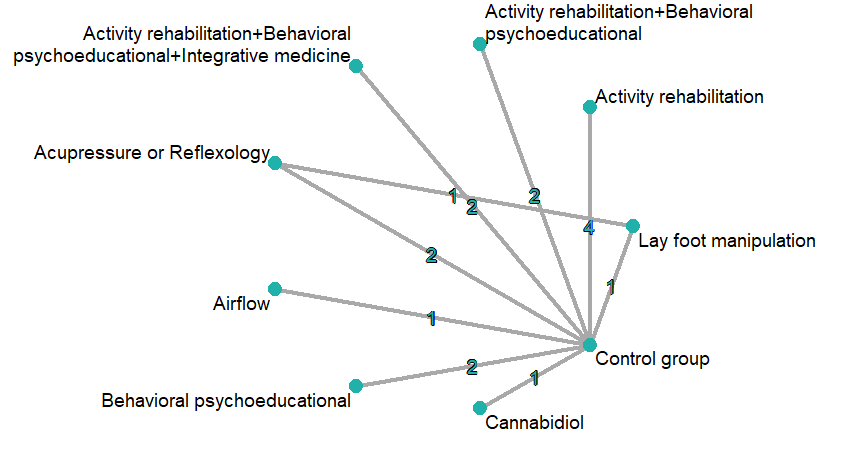


Nodes indicate different interventions or control groups and the number of studies contributing to direct comparisons. Numbers placed on each line correspond to the number of RCTs contributing to direct comparison.

# Appendix 4. Global Inconsistency of all outcomes

| **Outcome** | **Q-score** | **P-value** | **tau.within** | **tau2.within** |
| --- | --- | --- | --- | --- |
| Dyspnea Severity | 14.48 (df=1) | 0.0001 | 0.5042 | 0.2542 |
| Anxiety | 0.00 (df=0) | -- | 0.0905 | 0.0082 |
| Exercise Capacity | 0.00 (df=0) | -- | 61.5179 | 3784.4465 |
| Health-related Quality of Life | 30.56 (df=1) | < 0.0001 | 0.2817 | 0.0794 |

# Appendix 5. Local inconsistency in network


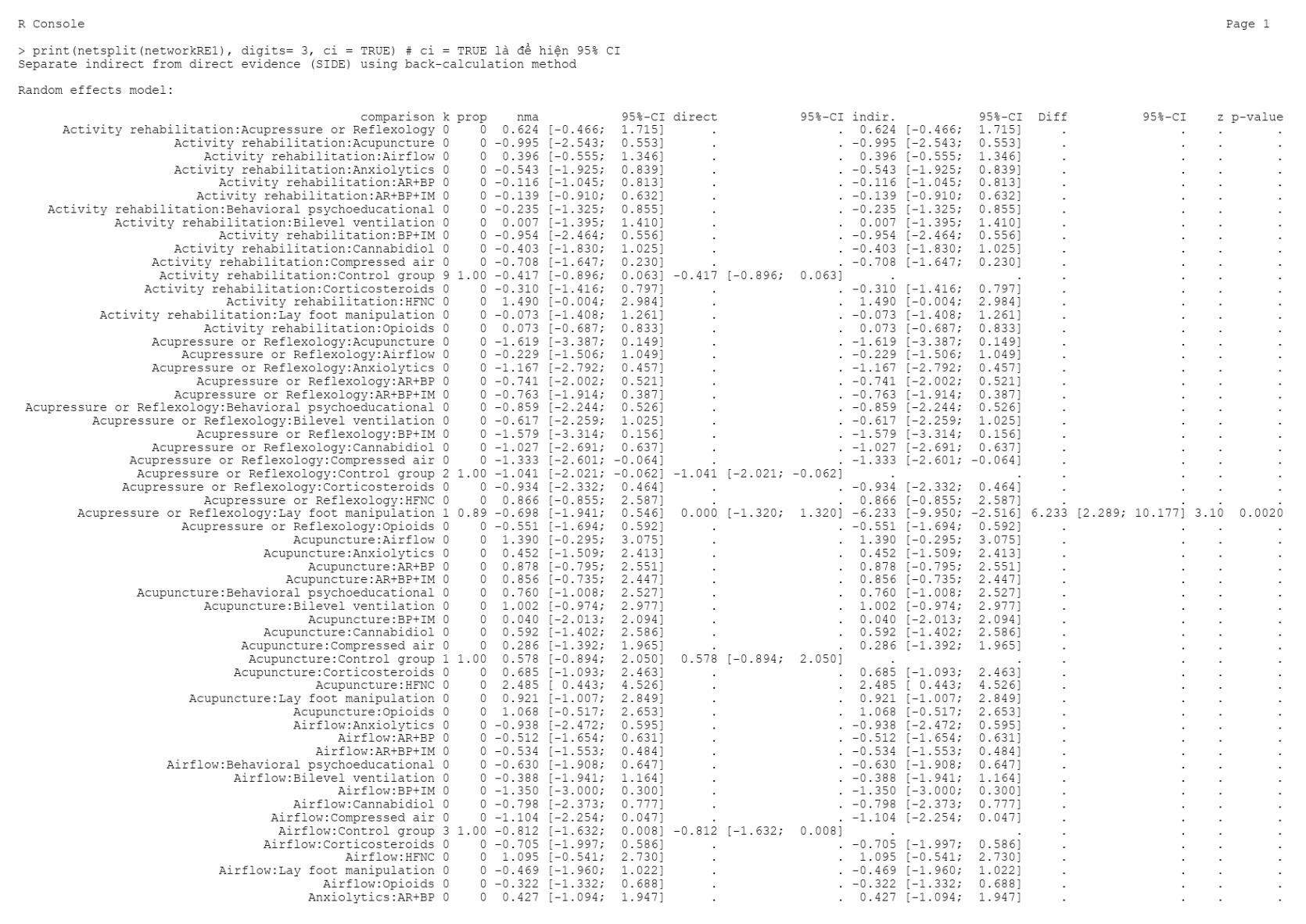
**Table 5.1 Outcome 1 - Dyspnea Severity**


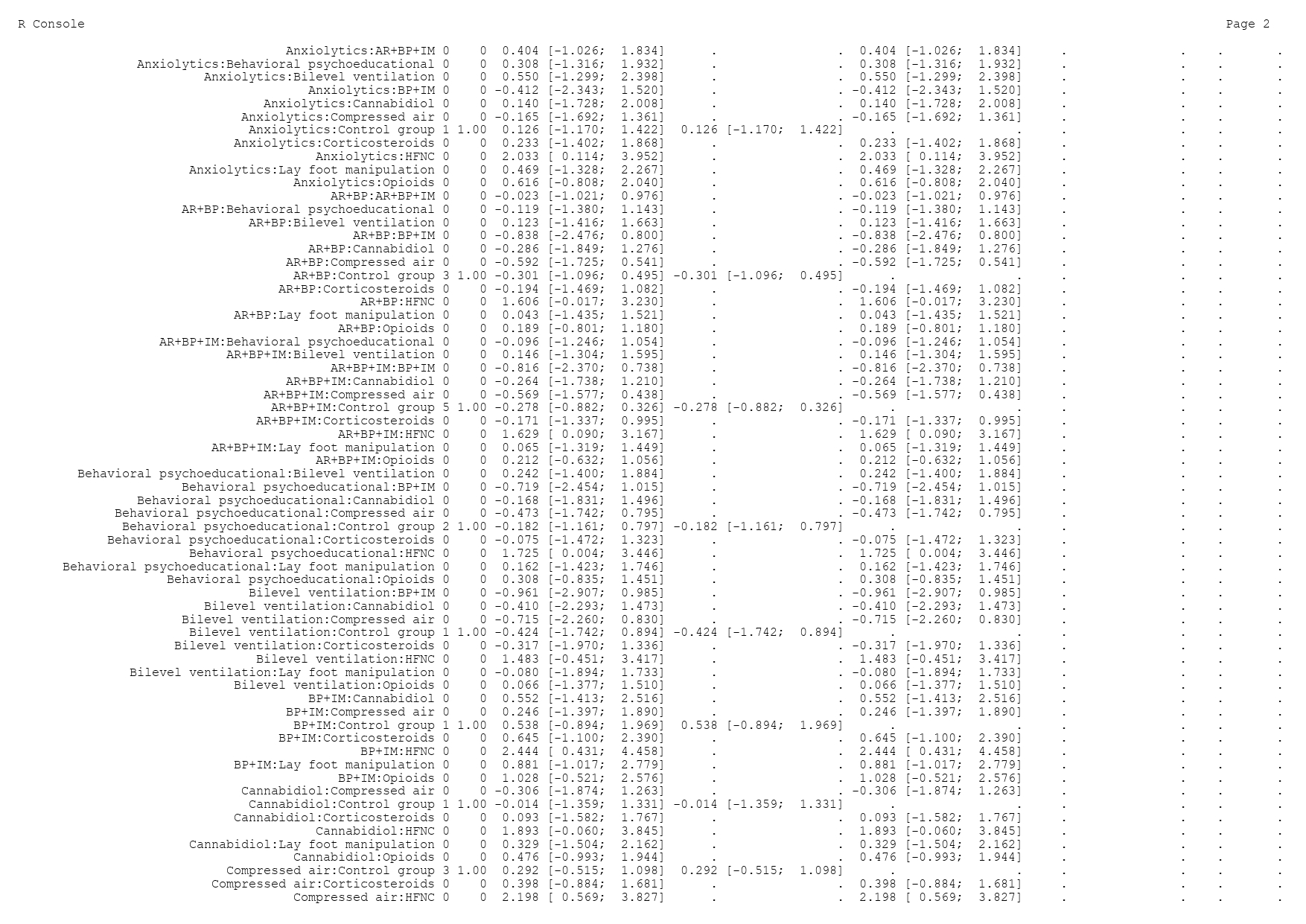


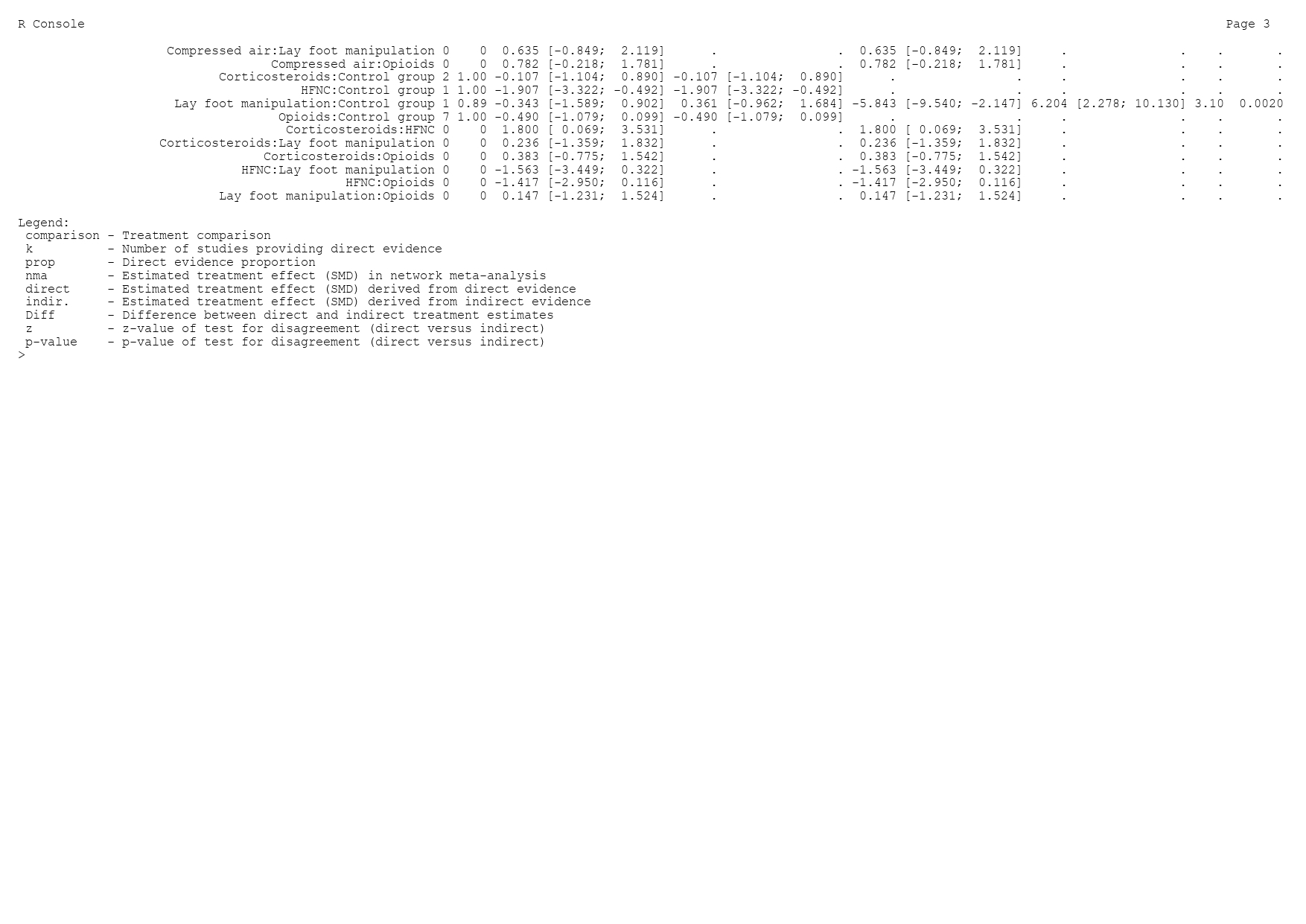


Legend:

comparison - Treatment comparison

k - Number of studies providing direct evidence

prop - Direct evidence proportion

nma - Estimated treatment effect (SMD) in network meta-analysis

direct - Estimated treatment effect (SMD) derived from direct evidence

indir. - Estimated treatment effect (SMD) derived from indirect evidence

Diff - Difference between direct and indirect treatment estimates

z - z-value of test for disagreement (direct versus indirect)

p-value - p-value of test for disagreement (direct versus indirect)

*Note: Activity rehabilitation+Behavioral psychoeducational = AR+BP; Activity rehabilitation+Behavioral psychoeducational+Integrative medicine = AR+BP+IM; Behavioral psychoeducational+Integrative medicine = BP+IM.*

**Table 5.2 Outcome 2 - Anxiety**

**
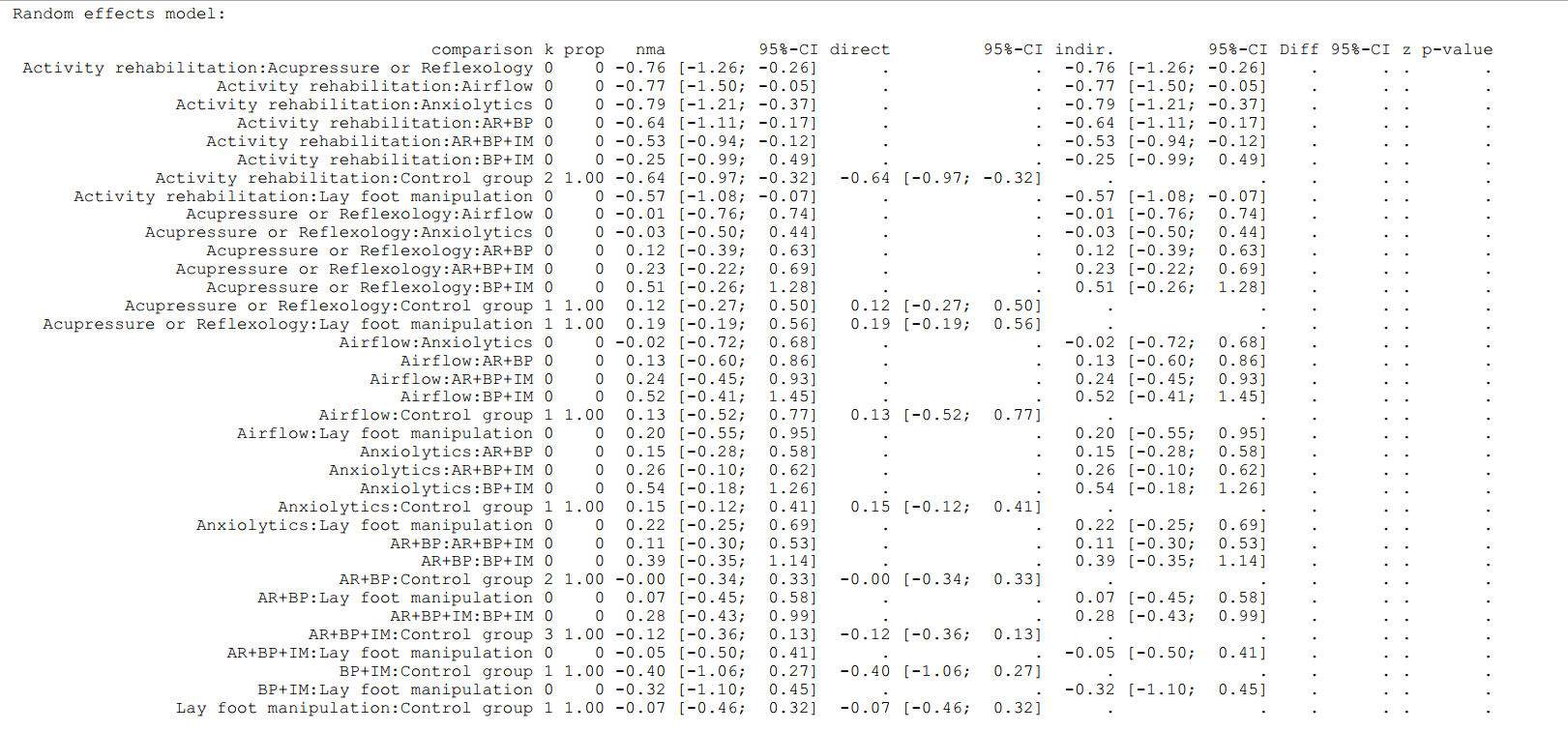
**

*Note: Activity rehabilitation+Behavioral psychoeducational = AR+BP; Activity rehabilitation+Behavioral psychoeducational+Integrative medicine = AR+BP+IM; Behavioral psychoeducational+Integrative medicine = BP+IM.*

**Table 5.3: Outcome 3 - Exercise Capacity**


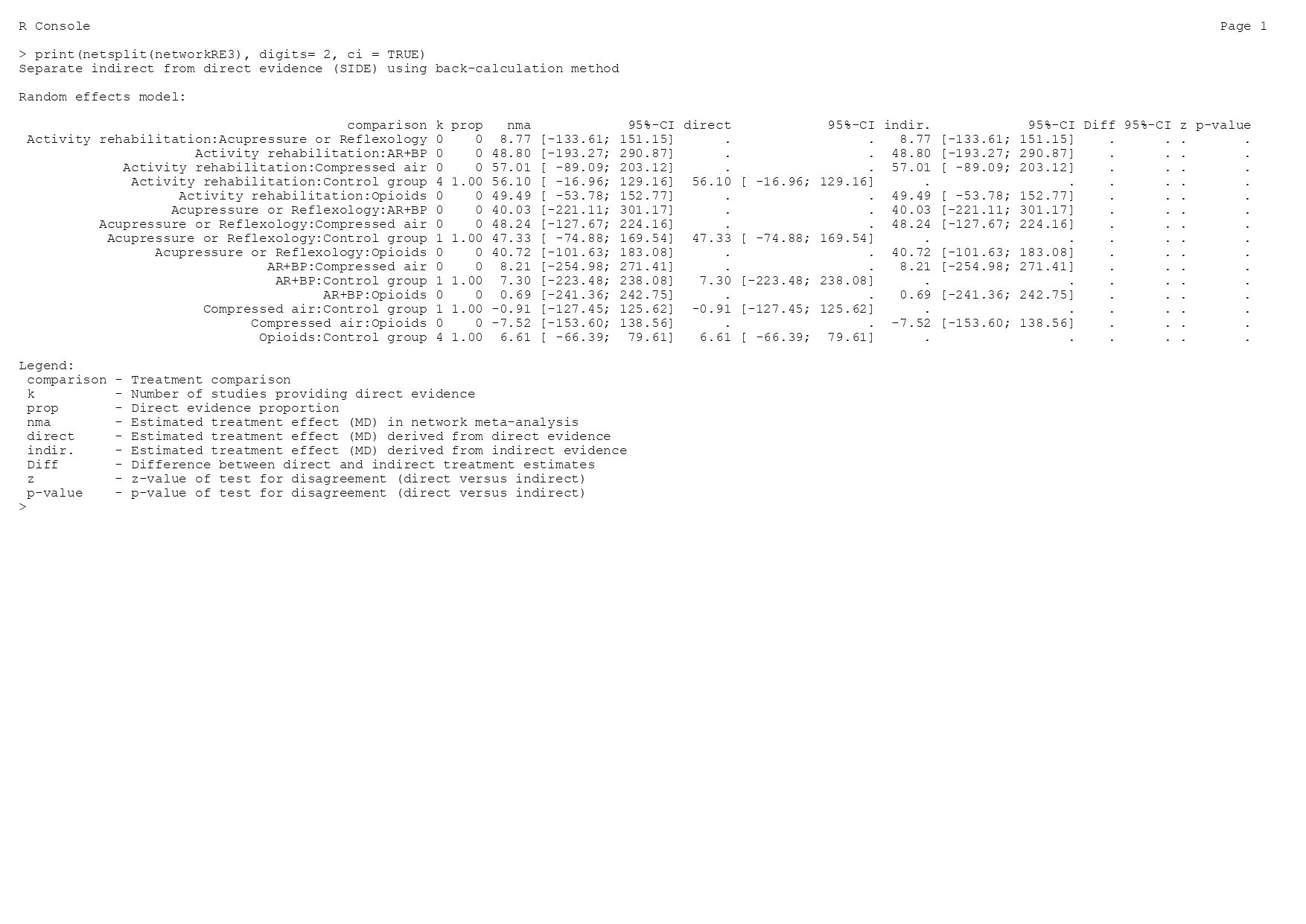


*Note: Activity rehabilitation+Behavioral psychoeducational = AR+BP; Activity rehabilitation+Behavioral psychoeducational+Integrative medicine = AR+BP+IM; Behavioral psychoeducational+Integrative medicine = BP+IM.*

**Table 5.4: Outcome 4 - Health-related Quality of Life**


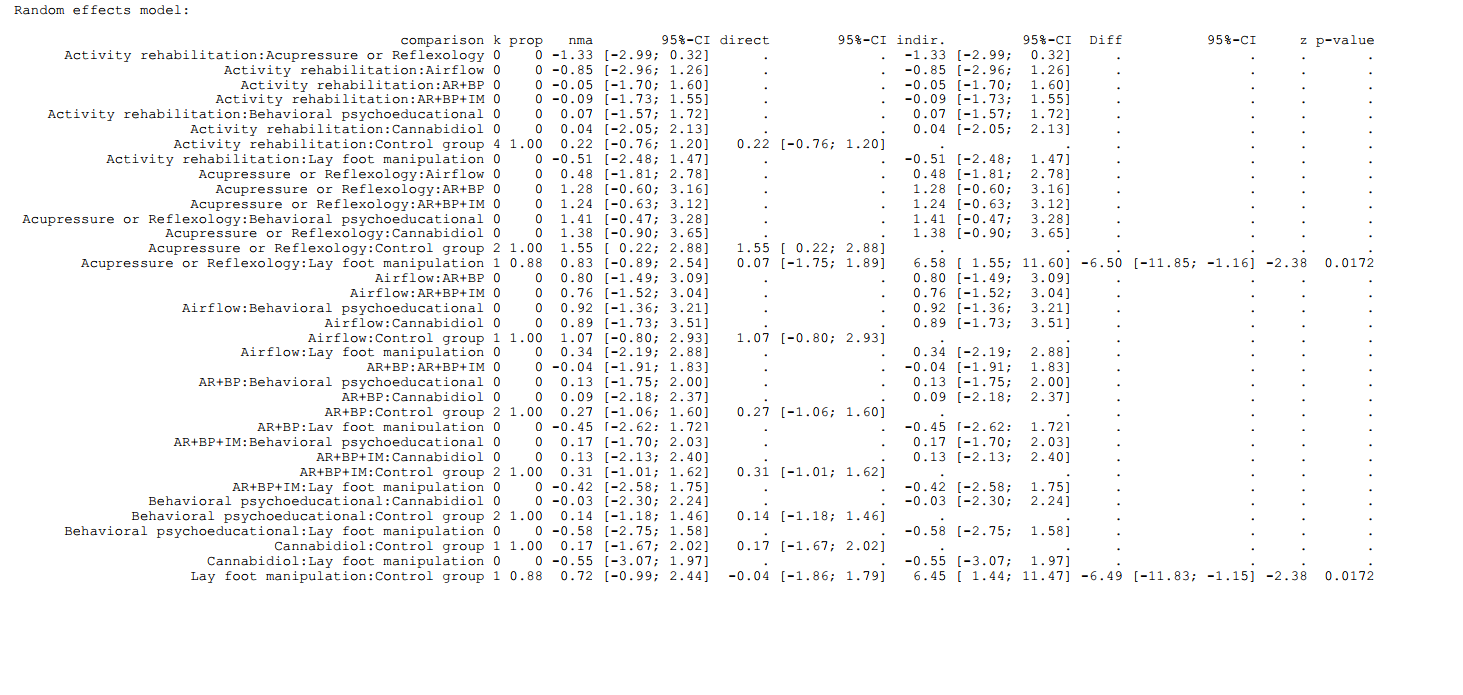


*Note: Activity rehabilitation+Behavioral psychoeducational = AR+BP; Activity rehabilitation+Behavioral psychoeducational+Integrative medicine = AR+BP+IM; Behavioral psychoeducational+Integrative medicine = BP+IM.*

**Appendix 6. Certainty of direct evidence assessment**

**Outcome 1: Dyspnea Severity**

| **Comparison** | **N of studies** | **Risk of bias** | **Inconsistency** | **Indirectness** | **Imprecision** | **Publication bias** | **SMD (95%CI)** | **Certainty of evidence** |
| --- | --- | --- | --- | --- | --- | --- | --- | --- |
| Activity rehabilitation: Control group | 9 | Serious | Serious | Very Serious | Serious | Unclear^1^ | -0.42 (-0.90; 0.06) | Very low |
| Acupressure or Reflexology: Control group | 2 | Not serious | Very Serious | Serious | Serious | Unclear^1^ | -1.04 (-2.02; -0.06) | Very low |
| Acupressure or Reflexology: Lay foot manipulation | 1 | Not serious | Serious | Not serious | Serious | Unclear^1^ | 0.00 (-1.32; 1.32) | Low |
| Acupuncture: Control group | 1 | Not serious | NA^2^ | Not serious | Very Serious | Unclear^1^ | 0.58 (-0.89; 2.05) | Low |
| Airflow: Control group | 3 | Not serious | Very Serious | Not serious | Very Serious | Unclear^1^ | -0.81 (-1.63; 0.01) | Very low |
| Anxiolytics: Control group | 1 | Not serious | NA^2^ | Not serious | Serious | Unclear^1^ | 0.13 (-1.17; 1.42) | Moderate |
| AR+BP: Control group | 3 | Serious | Not Serious | Serious | Very Serious | Unclear^1^ | -0.30 (-1.10; 0.50) | Very low |
| AR+BP+IM: Control group | 5 | Serious | Not Serious | Very Serious | Serious | Unclear^1^ | -0.28 (-0.88; 0.33) | Very low |
| Behavioral psychoeducational: Control group | 2 | Serious | Not Serious | Very Serious | Very Serious | Unclear^1^ | -0.18 (-1.16; 0.80) | Very low |
| Bilevel ventilation: Control group | 1 | Not serious | NA^2^ | Not serious | Serious | Unclear^1^ | -0.42 (-1.74; 0.89) | Moderate |
| BP+IM: Control group | 1 | Not serious | NA^2^ | Not serious | Very Serious | Unclear^1^ | 0.54 (-0.89; 1.97) | Low |
| Cannabidiol: Control group | 1 | Not serious | NA^2^ | Not serious | Very Serious | Unclear^1^ | -0.01 (-1.36; 1.33) | Low |
| Compressed air: Control group | 3 | Not serious | Not Serious | Not serious | Very Serious | Unclear^1^ | 0.29 (-0.52; 1.10) | Low |
| Corticosteroids: Control group | 2 | Not serious | Not Serious | Not serious | Very Serious | Unclear^1^ | -0.11 (-1.10; 0.89) | Low |
| HFNC: Control group | 1 | Not serious | NA^2^ | Not serious | Serious | Unclear^1^ | -1.91 (-3.32; -0.49) | Moderate |
| Lay foot manipulation: Control group | 1 | Not serious | Serious | Not serious | Serious | Unclear^1^ | 0.36 (-0.96; 1.68) | Low |
| Opioids: Control group | 7 | Not serious | Not Serious | Very Serious | Serious | Unclear^1^ | -0.49 (-1.08; 0.10) | Very low |

^1^ The funnel plot or Egger’s test was not performed because of insufficient information (<10 studies).

^2^ Unable to assess because there are <2 studies available with non-zero events in both arms.

**Outcome 2: Anxiety**

| **Comparison** | **N of studies** | **Risk of bias** | **Inconsistency** | **Indirectness** | **Imprecision** | **Publication bias** | **SMD**  **(95%CI)** | **Certainty of evidence** |
| --- | --- | --- | --- | --- | --- | --- | --- | --- |
| Activity rehabilitation: Control group | 2 | Not serious | Serious | Very Serious | No Serious | Unclear^1^ | -0.64 (-0.97; -0.32) | Very low |
| Acupressure or Reflexology: Control group | 1 | Not serious | NA^2^ | Not serious | Serious | Unclear^1^ | 0.12 (-0.27; 0.50) | Moderate |
| Acupressure or Reflexology: Lay foot manipulation | 1 | Not serious | NA^2^ | Not serious | Serious | Unclear^1^ | 0.19 (-0.19; 0.56) | Moderate |
| Airflow: Control group | 1 | Not serious | NA^2^ | Not serious | Serious | Unclear^1^ | 0.13 (-0.52; 0.77) | Moderate |
| Anxiolytics: Control group | 1 | Not serious | NA^2^ | Not serious | Serious | Unclear^1^ | 0.15 (-0.12; 0.41) | Moderate |
| AR+BP: Control group | 2 | Serious | Not serious | Serious | Serious | Unclear^1^ | -0.00 (-0.34; 0.33) | Very low |
| AR+BP+IM: Control group | 3 | Serious | Not serious | Very Serious | Serious | Unclear^1^ | -0.12 (-0.36; 0.13) | Very low |
| BP+IM: Control group | 1 | Not serious | NA^2^ | Not serious | Very Serious | Unclear^1^ | -0.40 (-1.06; 0.27) | Low |
| Lay foot manipulation: Control group | 1 | Not serious | NA^2^ | Not serious | Serious | Unclear^1^ | -0.07 (-0.46; 0.32) | Moderate |

Note: Activity rehabilitation+Behavioral psychoeducational = AR+BP; Activity rehabilitation+Behavioral psychoeducational+Integrative medicine = AR+BP+IM; Behavioral psychoeducational+Integrative medicine = BP+IM.

^1^ The funnel plot or Egger’s test was not performed because of insufficient information (<10 studies).

^2^ Unable to assess because there are <2 studies available with non-zero events in both arms.

**Outcome 3: Exercise Capacity**

| **Comparison** | **N of studies** | **Risk of bias** | **Inconsistency** | **Indirectness** | **Imprecision** | **Publication bias** | **MD (95%CI)** | **Certainty of evidence** |
| --- | --- | --- | --- | --- | --- | --- | --- | --- |
| Activity rehabilitation: Control group | 4 | Serious | Serious | Not serious | Very serious | Unclear^1^ | 56.10 (-16.96; 129.16) | Very low |
| Acupressure or Reflexology: Control group | 1 | Not serious | NA^2^ | Not serious | Very serious | Unclear^1^ | 47.33 (-74.88; 169.54) | Low |
| AR+BP: Control group | 1 | Not serious | NA^2^ | Not serious | Very serious | Unclear^1^ | 7.30 (-223.48; 238.08) | Low |
| Compressed air: Control group | 1 | Not serious | NA^2^ | Not serious | Very serious | Unclear^1^ | -0.91 (-127.45; 125.62) | Low |
| Opioids: Control group | 4 | Not serious | Not serious | Not serious | Very serious | Unclear^1^ | 6.61 (-66.39; 79.61) | Low |

Note: Activity rehabilitation+Behavioral psychoeducational = AR+BP

^1^ The funnel plot or Egger’s test was not performed because of insufficient information (<10 studies).

^2^ Unable to assess because there are <2 studies available with non-zero events in both arms.

**Outcome 4: Health-Related Quality of Life**

| **Comparison** | **N of studies** | **Risk of bias** | **Inconsistency** | **Indirectness** | **Imprecision** | **Publication bias** | **SMD (95%CI)** | **Certainty of evidence** |
| --- | --- | --- | --- | --- | --- | --- | --- | --- |
| Activity rehabilitation:Control group | 4 | Serious | Serious | Not serious | Serious | Unclear^1^ | 0.22 (-0.76; 1.20) | Very low |
| Acupressure or Reflexology:Control group | 2 | Not serious | Serious | Not serious | Not serious | Unclear^1^ | 1.55 (0.22; 2.88) | Moderate |
| Acupressure or Reflexology:Lay foot manipulation | 1 | Not serious | Serious | Not serious | Serious | Unclear^1^ | 0.07 (-1.75; 1.89) | Low |
| Airflow:Control group | 1 | Serious | NA^2^ | Not serious | Serious | Unclear^1^ | 1.07 (-0.80; 2.93) | Low |
| AR+BP:Control group | 2 | Not serious | Not serious | Not serious | Serious | Unclear^1^ | 0.27 (-1.06; 1.60) | Moderate |
| AR+BP+IM:Control group | 2 | Not serious | Not serious | Not serious | Serious | Unclear^1^ | 0.31 (-1.01; 1.62) | Moderate |
| Behavioral psychoeducational:Control group | 2 | Serious | Not serious | Not serious | Serious | Unclear^1^ | 0.14 (-1.18; 1.46) | Low |
| Cannabidiol:Control group | 1 | Not serious | NA^2^ | Not serious | Serious | Unclear^1^ | 0.17 (-1.67; 2.02) | Moderate |
| Lay foot manipulation:Control group | 1 | Not serious | Serious | Not serious | Serious | Unclear^1^ | -0.04 (-1.86; 1.79) | Low |

Note: Activity rehabilitation+Behavioral psychoeducational = AR+BP; Activity rehabilitation+Behavioral psychoeducational+Integrative medicine = AR+BP+IM

^1^ The funnel plot or Egger’s test was not performed because of insufficient information (<10 studies).

^2^ Unable to assess because there are <2 studies available with non-zero events in both arms.

# Appendix 7. Certainty of network evidence assessment

**Outcome 1: Dyspnea Severity**

| **Comparison** | **Direct evidence** | | **Indirect evidence** | | **Network Meta-analysis** | |
| --- | --- | --- | --- | --- | --- | --- |
|  | **SMD (95%CI)** | **Certainty of evidence** | **SMD (95%CI)** | **Certainty of evidence** | **SMD (95%CI)** | **Certainty of evidence** |
| Activity rehabilitation:Acupressure or Reflexology | **-** | **-** | 0.62 (-0.47; 1.72) | Very low^1^ | 0.62 (-0.47; 1.72) | Very low^7^ |
| Activity rehabilitation:Acupuncture | **-** | **-** | -0.99 (-2.54; 0.55) | Very low^1^ | -0.99 (-2.54; 0.55) | Very low^7^ |
| Activity rehabilitation:Airflow | **-** | **-** | 0.4 (-0.55; 1.35) | Very low^1^ | 0.4 (-0.55; 1.35) | Very low^7^ |
| Activity rehabilitation:Anxiolytics | **-** | **-** | -0.54 (-1.92; 0.84) | Very low^1^ | -0.54 (-1.92; 0.84) | Very low^7^ |
| Activity rehabilitation:AR+BP | **-** | **-** | -0.12 (-1.05; 0.81) | Very low^1^ | -0.12 (-1.05; 0.81) | Very low^7^ |
| Activity rehabilitation:AR+BP+IM | **-** | **-** | -0.14 (-0.91; 0.63) | Very low^1^ | -0.14 (-0.91; 0.63) | Very low^7^ |
| Activity rehabilitation:Behavioral psychoeducational | **-** | **-** | -0.23 (-1.33; 0.86) | Very low^1^ | -0.23 (-1.33; 0.86) | Very low^7^ |
| Activity rehabilitation:Bilevel ventilation | **-** | **-** | 0.01 (-1.40; 1.41) | Very low^1^ | 0.01 (-1.40; 1.41) | Very low^7^ |
| Activity rehabilitation:BP+IM | **-** | **-** | -0.95 (-2.46; 0.56) | Very low^1^ | -0.95 (-2.46; 0.56) | Very low^7^ |
| Activity rehabilitation:Cannabidiol | **-** | **-** | -0.4 (-1.83; 1.03) | Very low^1^ | -0.4 (-1.83; 1.03) | Very low^7^ |
| Activity rehabilitation:Compressed air | **-** | **-** | -0.71 (-1.65; 0.23) | Very low^1^ | -0.71 (-1.65; 0.23) | Very low^7^ |
| Activity rehabilitation:Control group | -0.42 (-0.90; 0.06) | Very low | **-** | **-** | -0.42 (-0.90; 0.06) | Very low^6^ |
| Activity rehabilitation:Corticosteroids | **-** | **-** | -0.31 (-1.42; 0.80) | Very low^1^ | -0.31 (-1.42; 0.80) | Very low^7^ |
| Activity rehabilitation:HFNC | **-** | **-** | 1.49 (0.00; 2.98) | Very low^1^ | 1.49 (0.00; 2.98) | Very low^7^ |
| Activity rehabilitation:Lay foot manipulation | **-** | **-** | -0.07 (-1.41; 1.26) | Very low^1^ | -0.07 (-1.41; 1.26) | Very low^7^ |
| Activity rehabilitation:Opioids | **-** | **-** | 0.07 (-0.69; 0.83) | Very low^1^ | 0.07 (-0.69; 0.83) | Very low^7^ |
| Acupressure or Reflexology:Acupuncture | **-** | **-** | -1.62 (-3.39; 0.15) | Very low^1^ | -1.62 (-3.39; 0.15) | Very low^7^ |
| Acupressure or Reflexology:Airflow | **-** | **-** | -0.23 (-1.51; 1.05) | Very low^2^ | -0.23 (-1.51; 1.05) | Very low^7^ |
| Acupressure or Reflexology:Anxiolytics | **-** | **-** | -1.17 (-2.79; .46) | Very low^1^ | -1.17 (-2.79; 0.46) | Very low^7^ |
| Acupressure or Reflexology:AR+BP | **-** | **-** | -0.74 (-2.00; 0.52) | Very low^2^ | -0.74 (-2.00; 0.52) | Very low^7^ |
| Acupressure or Reflexology:AR+BP+IM | **-** | **-** | -0.76 (-1.91; 0.39) | Very low^2^ | -0.76 (-1.91; 0.39) | Very low^7^ |
| Acupressure or Reflexology:Behavioral psychoeducational | **-** | **-** | -0.86 (-2.24; 0.53) | Very low^2^ | -0.86 (-2.24; 0.53) | Very low^7^ |
| Acupressure or Reflexology:Bilevel ventilation | **-** | **-** | -0.62 (-2.26; 1.02) | Very low^1^ | -0.62 (-2.26; 1.02) | Very low^7^ |
| Acupressure or Reflexology:BP+IM | **-** | **-** | -1.58 (-3.31; 0.16) | Very low^1^ | -1.58 (-3.31; 0.16) | Very low^7^ |
| Acupressure or Reflexology:Cannabidiol | **-** | **-** | -1.03 (-2.69; 0.64) | Very low^1^ | -1.03 (-2.69; 0.64) | Very low^7^ |
| Acupressure or Reflexology:Compressed air | **-** | **-** | -1.33 (-2.60; -0.06) | Very low^1^ | -1.33 (-2.60; -0.06) | Very low |
| Acupressure or Reflexology:Control group | -1.04 (-2.02; -0.06) | Very low | **-** | **-** | -1.04 (-2.02; -0.06) | Very low^6^ |
| Acupressure or Reflexology:Corticosteroids | **-** | **-** | -0.93 (-2.33; 0.46) | Very low^1^ | -0.93 (-2.33; 0.46) | Very low^7^ |
| Acupressure or Reflexology:HFNC | **-** | **-** | 0.87 (-0.86; 2.59) | Very low^1^ | 0.87 (-0.86; 2.59) | Very low^7^ |
| Acupressure or Reflexology:Lay foot manipulation | 0.00 (-1.32; 1.32) | Low | -6.23 (-9.95; -2.52) | Very low^1^ | -0.7 (-1.94; 0.55) | Low^6,7^ |
| Acupressure or Reflexology:Opioids | **-** | **-** | -0.55 (-1.69; 0.59) | Very low^2^ | -0.55 (-1.69; 0.59) | Very low^7^ |
| Acupuncture:Airflow | **-** | **-** | 1.39 (-0.29; 3.07) | Very low^1^ | 1.39 (-0.29; 3.07) | Very low^7^ |
| Acupuncture:Anxiolytics | **-** | **-** | 0.45 (-1.51; 2.41) | Low^3^ | 0.45 (-1.51; 2.41) | Very low^7^ |
| Acupuncture:AR+BP | **-** | **-** | 0.88 (-0.79; 2.55) | Very low^1^ | 0.88 (-0.79; 2.55) | Very low^7^ |
| Acupuncture:AR+BP+IM | **-** | **-** | 0.86 (-0.73; 2.45) | Very low^1^ | 0.86 (-0.73; 2.45) | Very low^7^ |
| Acupuncture:Behavioral psychoeducational | **-** | **-** | 0.76 (-1.01; 2.53) | Very low^1^ | 0.76 (-1.01; 2.53) | Very low^7^ |
| Acupuncture:Bilevel ventilation | **-** | **-** | 1 (-0.97; 2.98) | Low^3^ | 1 (-0.97; 2.98) | Very low^7^ |
| Acupuncture:BP+IM | **-** | **-** | 0.04 (-2.01; 2.09) | Low^4^ | 0.04 (-2.01; 2.09) | Very low^7^ |
| Acupuncture:Cannabidiol | **-** | **-** | 0.59 (-1.40; 2.59) | Low^4^ | 0.59 (-1.40; 2.59) | Very low^7^ |
| Acupuncture:Compressed air | **-** | **-** | 0.29 (-1.39; 1.96) | Low^4^ | 0.29 (-1.39; 1.96) | Very low^7^ |
| Acupuncture:Control group | 0.58 (-0.89; 2.05) | Low | **-** | **-** | 0.58 (-0.89; 2.05) | Very low^6,7^ |
| Acupuncture:Corticosteroids | **-** | **-** | 0.68 (-1.09; 2.46) | Low^4^ | 0.68 (-1.09; 2.46) | Very low^7^ |
| Acupuncture:HFNC | **-** | **-** | 2.48 (0.44; 4.53) | Low^3^ | 2.48 (0.44; 4.53) | Low |
| Acupuncture:Lay foot manipulation | **-** | **-** | 0.92 (-1.01; 2.85) | Low^3^ | 0.92 (-1.01; 2.85) | Very low^7^ |
| Acupuncture:Opioids | **-** | **-** | 1.07 (-0.52; 2.65) | Very low^1^ | 1.07 (-0.52; 2.65) | Very low^7^ |
| Airflow:Anxiolytics | **-** | **-** | -0.94 (-2.47; 0.60) | Very low^1^ | -0.94 (-2.47; 0.60) | Very low^7^ |
| Airflow:AR+BP | **-** | **-** | -0.51 (-1.65; 0.63) | Very low^2^ | -0.51 (-1.65; 0.63) | Very low^7^ |
| Airflow:AR+BP+IM | **-** | **-** | -0.53 (-1.55; 0.48) | Very low^2^ | -0.53 (-1.55; 0.48) | Very low^7^ |
| Airflow:Behavioral psychoeducational | **-** | **-** | -0.63 (-1.91; 0.65) | Very low^2^ | -0.63 (-1.91; 0.65) | Very low^7^ |
| Airflow:Bilevel ventilation | **-** | **-** | -0.39 (-1.94; 1.16) | Very low^1^ | -0.39 (-1.94; 1.16) | Very low^7^ |
| Airflow:BP+IM | **-** | **-** | -1.35 (-3.00; 0.30) | Very low^1^ | -1.35 (-3.00; 0.30) | Very low^7^ |
| Airflow:Cannabidiol | **-** | **-** | -0.80 (-2.37; 0.78) | Very low^1^ | -0.8 (-2.37; 0.78) | Very low^7^ |
| Airflow:Compressed air | **-** | **-** | -1.10 (-2.25; 0.05) | Very low^1^ | -1.1 (-2.25; 0.05) | Very low^7^ |
| Airflow:Control group | -0.81 (-1.63; 0.01) | Very low | **-** | **-** | -0.81 (-1.63; 0.01) | Very low^67^ |
| Airflow:Corticosteroids | **-** | **-** | -0.71 (-2.00; 0.59) | Very low^1^ | -0.71 (-2.00; 0.59) | Very low^7^ |
| Airflow:HFNC | **-** | **-** | 1.09 (-0.54; 2.73) | Very low^1^ | 1.09 (-0.54; 2.73) | Very low^7^ |
| Airflow:Lay foot manipulation | **-** | **-** | -0.47 (-1.96; 1.02) | Very low^1^ | -0.47 (-1.96; 1.02) | Very low^7^ |
| Airflow:Opioids | **-** | **-** | -0.32 (-1.33; 0.69) | Very low^2^ | -0.32 (-1.33; 0.69) | Very low^7^ |
| Anxiolytics:AR+BP | **-** | **-** | 0.43 (-1.09; 1.95) | Very low^1^ | 0.43 (-1.09; 1.95) | Very low^7^ |
| Anxiolytics:AR+BP+IM | **-** | **-** | 0.40 (-1.03; 1.83) | Very low^1^ | 0.4 (-1.03; 1.83) | Very low^7^ |
| Anxiolytics:Behavioral psychoeducational | **-** | **-** | 0.31 (-1.32; 1.93) | Very low^1^ | 0.31 (-1.32; 1.93) | Very low^7^ |
| Anxiolytics:Bilevel ventilation | **-** | **-** | 0.55 (-1.30; 2.40) | Moderate^5^ | 0.55 (-1.30; 2.40) | Low^7^ |
| Anxiolytics:BP+IM | **-** | **-** | -0.41 (-2.34; 1.52) | Low^3^ | -0.41 (-2.34; 1.52) | Very low^7^ |
| Anxiolytics:Cannabidiol | **-** | **-** | 0.14 (-1.73; 2.01) | Low^3^ | 0.14 (-1.73; 2.01) | Very low^7^ |
| Anxiolytics:Compressed air | **-** | **-** | -0.17 (-1.69; 1.36) | Low^3^ | -0.17 (-1.69; 1.36) | Very low^7^ |
| Anxiolytics:Control group | 0.13 (-1.17; 1.42) | Moderate | **-** | **-** | 0.13 (-1.17; 1.42) | Low^6,7^ |
| Anxiolytics:Corticosteroids | **-** | **-** | 0.23 (-1.40; 1.87) | Low^3^ | 0.23 (-1.40; 1.87) | Very low^7^ |
| Anxiolytics:HFNC | **-** | **-** | 2.03 (0.11; 3.95) | Moderate^5^ | 2.03 (0.11; 3.95) | Moderate |
| Anxiolytics:Lay foot manipulation | **-** | **-** | 0.47 (-1.33; 2.27) | Moderate^5^ | 0.47 (-1.33; 2.27) | Low^7^ |
| Anxiolytics:Opioids | **-** | **-** | 0.62 (-0.81; 2.04) | Very low^1^ | 0.62 (-0.81; 2.04) | Very low^7^ |
| AR+BP:AR+BP+IM | **-** | **-** | -0.02 (-1.02; 0.98) | Very low^2^ | -0.02 (-1.02; 0.98) | Very low^7^ |
| AR+BP:Behavioral psychoeducational | **-** | **-** | -0.12 (-1.38; 1.14) | Very low^2^ | -0.12 (-1.38; 1.14) | Very low^7^ |
| AR+BP:Bilevel ventilation | **-** | **-** | 0.12 (-1.42; 1.66) | Very low^1^ | 0.12 (-1.42; 1.66) | Very low^7^ |
| AR+BP:BP+IM | **-** | **-** | -0.84 (-2.48; 0.80) | Very low^1^ | -0.84 (-2.48; 0.80) | Very low^7^ |
| AR+BP:Cannabidiol | **-** | **-** | -0.29 (-1.85; 1.28) | Very low^1^ | -0.29 (-1.85; 1.28) | Very low^7^ |
| AR+BP:Compressed air | **-** | **-** | -0.59 (-1.72; 0.54) | Very low^1^ | -0.59 (-1.72; 0.54) | Very low^7^ |
| AR+BP:Control group | -0.30 (-1.10; 0.50) | Very low | **-** | **-** | -0.3 (-1.10; 0.50) | Very low^6,7^ |
| AR+BP:Corticosteroids | **-** | **-** | -0.19 (-1.47; 1.08) | Very low^1^ | -0.19 (-1.47; 1.08) | Very low^7^ |
| AR+BP:HFNC | **-** | **-** | 1.61 (-0.02; 3.23) | Very low^1^ | 1.61 (-0.02; 3.23) | Very low^7^ |
| AR+BP:Lay foot manipulation | **-** | **-** | 0.04 (-1.44; 1.52) | Very low^1^ | 0.04 (-1.44; 1.52) | Very low^7^ |
| AR+BP:Opioids | **-** | **-** | 0.19 (-0.80; 1.18) | Very low^2^ | 0.19 (-0.80; 1.18) | Very low^7^ |
| AR+BP+IM:Behavioral psychoeducational | **-** | **-** | -0.1 (-1.25; 1.05) | Very low^2^ | -0.1 (-1.25; 1.05) | Very low^7^ |
| AR+BP+IM:Bilevel ventilation | **-** | **-** | 0.15 (-1.30; 1.60) | Very low^1^ | 0.15 (-1.30; 1.60) | Very low^7^ |
| AR+BP+IM:BP+IM | **-** | **-** | -0.82 (-2.37; 0.74) | Very low^1^ | -0.82 (-2.37; 0.74) | Very low^7^ |
| AR+BP+IM:Cannabidiol | **-** | **-** | -0.26 (-1.74; 1.21) | Very low^1^ | -0.26 (-1.74; 1.21) | Very low^7^ |
| AR+BP+IM:Compressed air | **-** | **-** | -0.57 (-1.58; 0.44) | Very low^1^ | -0.57 (-1.58; 0.44) | Very low^7^ |
| AR+BP+IM:Control group | -0.28 (-0.88; 0.33) | Very low | **-** | **-** | -0.28 (-0.88; 0.33) | Very low^6,7^ |
| AR+BP+IM:Corticosteroids | **-** | **-** | -0.17 (-1.34; 0.99) | Very low^1^ | -0.17 (-1.34; 0.99) | Very low^7^ |
| AR+BP+IM:HFNC | **-** | **-** | 1.63 ( 0.09; 3.17) | Very low^1^ | 1.63 (0.09; 3.17) | Very low^7^ |
| AR+BP+IM:Lay foot manipulation | **-** | **-** | 0.07 (-1.32; 1.45) | Very low^1^ | 0.07 (-1.32; 1.45) | Very low^7^ |
| AR+BP+IM:Opioids | **-** | **-** | 0.21 (-0.63; 1.06) | Very low^2^ | 0.21 (-0.63; 1.06) | Very low^7^ |
| Behavioral psychoeducational:Bilevel ventilation | **-** | **-** | 0.24 (-1.40; 1.88) | Very low^1^ | 0.24 (-1.40; 1.88) | Very low^7^ |
| Behavioral psychoeducational:BP+IM | **-** | **-** | -0.72 (-2.45; 1.02) | Very low^1^ | -0.72 (-2.45; 1.02) | Very low^7^ |
| Behavioral psychoeducational:Cannabidiol | **-** | **-** | -0.17 (-1.83; 1.50) | Very low^1^ | -0.17 (-1.83; 1.50) | Very low^7^ |
| Behavioral psychoeducational:Compressed air | **-** | **-** | -0.47 (-1.74; 0.80) | Very low^1^ | -0.47 (-1.74; 0.80) | Very low^7^ |
| Behavioral psychoeducational:Control group | -0.18 (-1.16; 0.80) | Very low | **-** | **-** | -0.18 (-1.16; 0.80) | Very low^6,7^ |
| Behavioral psychoeducational:Corticosteroids | **-** | **-** | -0.07 (-1.47; 1.32) | Very low^1^ | -0.07 (-1.47; 1.32) | Very low^7^ |
| Behavioral psychoeducational:HFNC | **-** | **-** | 1.72 (0.00; 3.45) | Very low^1^ | 1.72 (0.00; 3.45) | Very low^7^ |
| Behavioral psychoeducational:Lay foot manipulation | **-** | **-** | 0.16 (-1.42; 1.75) | Very low^1^ | 0.16 (-1.42; 1.75) | Very low^7^ |
| Behavioral psychoeducational:Opioids | **-** | **-** | 0.31 (-0.83; 1.45) | Very low^2^ | 0.31 (-0.83; 1.45) | Very low^7^ |
| Bilevel ventilation:BP+IM | **-** | **-** | -0.96 (-2.91; 0.98) | Low^3^ | -0.96 (-2.91; 0.98) | Very low^7^ |
| Bilevel ventilation:Cannabidiol | **-** | **-** | -0.41 (-2.29; 1.47) | Low^3^ | -0.41 (-2.29; 1.47) | Very low^7^ |
| Bilevel ventilation:Compressed air | **-** | **-** | -0.72 (-2.26; 0.83) | Low^3^ | -0.72 (-2.26; 0.83) | Very low^7^ |
| Bilevel ventilation:Control group | -0.42 (-1.74; 0.89) | Moderate | **-** | **-** | -0.42 (-1.74; 0.89) | Low^6,7^ |
| Bilevel ventilation:Corticosteroids | **-** | **-** | -0.32 (-1.97; 1.34) | Low^3^ | -0.32 (-1.97; 1.34) | Very low^7^ |
| Bilevel ventilation:HFNC | **-** | **-** | 1.48 (-0.45; 3.42) | Moderate^5^ | 1.48 (-0.45; 3.42) | Low^7^ |
| Bilevel ventilation:Lay foot manipulation | **-** | **-** | -0.08 (-1.89; 1.73) | Moderate^5^ | -0.08 (-1.89; 1.73) | Low^7^ |
| Bilevel ventilation:Opioids | **-** | **-** | 0.07 (-1.38; 1.51) | Very low^1^ | 0.07 (-1.38; 1.51) | Very low^7^ |
| BP+IM:Cannabidiol | **-** | **-** | 0.55 (-1.41; 2.52) | Low^4^ | 0.55 (-1.41; 2.52) | Very low^7^ |
| BP+IM:Compressed air | **-** | **-** | 0.25 (-1.40; 1.89) | Low^4^ | 0.25 (-1.40; 1.89) | Very low^7^ |
| BP+IM:Control group | 0.54 (-0.89; 1.97) | Low | **-** | **-** | 0.54 (-0.89; 1.97) | Very low^6,7^ |
| BP+IM:Corticosteroids | **-** | **-** | 0.64 (-1.10; 2.39) | Low^4^ | 0.64 (-1.10; 2.39) | Very low^7^ |
| BP+IM:HFNC | **-** | **-** | 2.44 (0.43; 4.46) | Low^3^ | 2.44 (0.43; 4.46) | Low |
| BP+IM:Lay foot manipulation | **-** | **-** | 0.88 (-1.02; 2.78) | Low^3^ | 0.88 (-1.02; 2.78) | Very low^7^ |
| BP+IM:Opioids | **-** | **-** | 1.03 (-0.52; 2.58) | Very low^1^ | 1.03 (-0.52; 2.58) | Very low^7^ |
| Cannabidiol:Compressed air | **-** | **-** | -0.31 (-1.87; 1.26) | Low^4^ | -0.31 (-1.87; 1.26) | Very low^7^ |
| Cannabidiol:Control group | -0.01 (-1.36; 1.33) | Low | **-** | **-** | -0.01 (-1.36; 1.33) | Very low^6,7^ |
| Cannabidiol:Corticosteroids | **-** | **-** | 0.09 (-1.58; 1.77) | Low^4^ | 0.09 (-1.58; 1.77) | Very low^7^ |
| Cannabidiol:HFNC | **-** | **-** | 1.89 (-0.06; 3.84) | Low^3^ | 1.89 (-0.06; 3.84) | Very low^7^ |
| Cannabidiol:Lay foot manipulation | **-** | **-** | 0.33 (-1.50; 2.16) | Low^3^ | 0.33 (-1.50; 2.16) | Very low^7^ |
| Cannabidiol:Opioids | **-** | **-** | 0.48 (-0.99; 1.94) | Very low^1^ | 0.48 (-0.99; 1.94) | Very low^7^ |
| Compressed air:Control group | 0.29 (-0.52; 1.10) | Low | **-** | **-** | 0.29 (-0.52; 1.10) | Very low^6,7^ |
| Compressed air:Corticosteroids | **-** | **-** | 0.4 (-0.88; 1.68) | Low^4^ | 0.4 (-0.88; 1.68) | Very low^7^ |
| Compressed air:HFNC | **-** | **-** | 2.2 (0.57; 3.83) | Low^3^ | 2.2 (0.57; 3.83) | Low |
| Compressed air:Lay foot manipulation | **-** | **-** | 0.63 (-0.85; 2.12) | Low^3^ | 0.63 (-0.85; 2.12) | Very low^7^ |
| Compressed air:Opioids | **-** | **-** | 0.78 (-0.22; 1.78) | Very low^1^ | 0.78 (-0.22; 1.78) | Very low^7^ |
| Corticosteroids:Control group | -0.11 (-1.10; 0.89) | Low | **-** | **-** | -0.11 (-1.10; 0.89) | Very low^6,7^ |
| HFNC:Control group | -1.91 (-3.32; -0.49) | Moderate | **-** | **-** | -1.91 (-3.32; -0.49) | Moderate |
| Lay foot manipulation:Control group | 0.36 (-0.96; 1.68) | Low | -5.84 (-9.54; -2.15) | Very low^1^ | -0.34 (-1.59; 0.90) | Low^6,7^ |
| Opioids:Control group | -0.49 (-1.08; 0.10) | Very low |  |  | -0.49 (-1.08; 0.10) | Very low^6,7^ |
| Corticosteroids:HFNC | **-** | **-** | 1.8 (0.07; 3.53) | Low^3^ | 1.8 (0.07; 3.53) | Low |
| Corticosteroids:Lay foot manipulation | **-** | **-** | 0.24 (-1.36; 1.83) | Low^3^ | 0.24 (-1.36; 1.83) | Very low^7^ |
| Corticosteroids:Opioids | **-** | **-** | 0.38 (-0.78; 1.54) | Very low^1^ | 0.38 (-0.78; 1.54) | Very low^7^ |
| HFNC:Lay foot manipulation | **-** | **-** | -1.56 (-3.45; 0.32) | Moderate^5^ | -1.56 (-3.45; 0.32) | Low^7^ |
| HFNC:Opioids | **-** | **-** | -1.42 (-2.95; 0.12) | Very low^1^ | -1.42 (-2.95; 0.12) | Very low^7^ |
| Lay foot manipulation:Opioids | **-** | **-** | 0.15 (-1.23; 1.52) | Very low^1^ | 0.15 (-1.23; 1.52) | Very low^7^ |

Note: Activity rehabilitation+Behavioral psychoeducational = AR+BP; Activity rehabilitation+Behavioral psychoeducational+Integrative medicine = AR+BP+IM; Behavioral psychoeducational+Integrative medicine = BP+IM.

^1^ The lower confidence rating of the two direct comparisons is very low,

^2^ The confidence ratings for both direct comparisons are very low,

^3^ The lower confidence rating of the two direct comparisons is low,

^4^ The confidence ratings for both direct comparisons are low,

^5^ The confidence ratings for both direct comparisons are moderate,

^6^ The contribution of the direct evidence to the network estimate is much greater than that of the indirect evidence,

^7^ Imprecise (95% CI includes SMD of 0).

**Outcome 2: Anxiety**

| **Comparison** | **Direct evidence** | | **Indirect evidence** | | **Network Meta-analysis** | |
| --- | --- | --- | --- | --- | --- | --- |
|  | **SMD (95%CI)** | **Certainty of evidence** | **SMD (95%CI)** | **Certainty of evidence** | **SMD (95%CI)** | **Certainty of evidence** |
| Activity rehabilitation:Acupressure or Reflexology | **-** | **-** | -0.76 (-1.26; -0.26) | Very low^1^ | -0.76 (-1.26; -0.26) | Very low |
| Activity rehabilitation:Airflow | **-** | **-** | -0.77 (-1.50; -0.05) | Very low^1^ | -0.77 (-1.50; -0.05) | Very low |
| Activity rehabilitation:Anxiolytics | **-** | **-** | -0.79 (-1.21; -0.37) | Very low^1^ | -0.79 (-1.21; -0.37) | Very low |
| Activity rehabilitation:AR+BP | **-** | **-** | -0.64 (-1.11; -0.17) | Very low^2^ | -0.64 (-1.11; -0.17) | Very low |
| Activity rehabilitation:AR+BP+IM | **-** | **-** | -0.53 (-0.94; -0.12) | Very low^2^ | -0.53 (-0.94; -0.12) | Very low |
| Activity rehabilitation:BP+IM | **-** | **-** | -0.25 (-0.99; 0.49) | Very low^1^ | -0.25 (-0.99; 0.49) | Very low^7^ |
| Activity rehabilitation:Control group | -0.64 (-0.97; -0.32) | Very low | **-** | **-** | -0.64 (-0.97; -0.32) | Very low^6^ |
| Activity rehabilitation:Lay foot manipulation | **-** | **-** | -0.57 (-1.08; -0.07) | Very low^1^ | -0.57 (-1.08; -0.07) | Very low |
| Acupressure or Reflexology:Airflow | **-** | **-** | -0.01 (-0.76; 0.74) | Moderate^5^ | -0.01 (-0.76; 0.74) | Low^7^ |
| Acupressure or Reflexology:Anxiolytics | **-** | **-** | -0.03 (-0.50; 0.44) | Moderate^5^ | -0.03 (-0.50; 0.44) | Low^7^ |
| Acupressure or Reflexology:AR+BP | **-** | **-** | 0.12 (-0.39; 0.63) | Very low^1^ | 0.12 (-0.39; 0.63) | Very low^7^ |
| Acupressure or Reflexology:AR+BP+IM | **-** | **-** | 0.23 (-0.22; 0.69) | Very low^1^ | 0.23 (-0.22; 0.69) | Very low^7^ |
| Acupressure or Reflexology:BP+IM | **-** | **-** | 0.51 (-0.26; 1.28) | Low^3^ | 0.51 (-0.26; 1.28) | Very low^7^ |
| Acupressure or Reflexology:Control group | 0.12 (-0.27; 0.50) | Moderate | **-** | **-** | 0.12 (-0.27; 0.50) | Low^6,7^ |
| Acupressure or Reflexology:Lay foot manipulation | 0.19 (-0.19; 0.56) | Moderate | **-** | **-** | 0.19 (-0.19; 0.56) | Low^6,7^ |
| Airflow:Anxiolytics | **-** | **-** | -0.02 (-0.72; 0.68) | Moderate^5^ | -0.02 (-0.72; 0.68) | Low^7^ |
| Airflow:AR+BP | **-** | **-** | 0.13 (-0.60; 0.86) | Very low^1^ | 0.13 (-0.60; 0.86) | Very low^7^ |
| Airflow:AR+BP+IM | **-** | **-** | 0.24 (-0.45; 0.93) | Very low^1^ | 0.24 (-0.45; 0.93) | Very low^7^ |
| Airflow:BP+IM | **-** | **-** | 0.52 (-0.41; 1.45) | Low^3^ | 0.52 (-0.41; 1.45) | Very low^7^ |
| Airflow:Control group | 0.13 (-0.52; 0.77) | Moderate | **-** | **-** | 0.13 (-0.52; 0.77) | Low^6,7^ |
| Airflow:Lay foot manipulation | **-** | **-** | 0.2 (-0.55; 0.95) | Moderate^5^ | 0.2 (-0.55; 0.95) | Low^7^ |
| Anxiolytics:AR+BP | **-** | **-** | 0.15 (-0.28; 0.58) | Very low^1^ | 0.15 (-0.28; 0.58) | Very low^7^ |
| Anxiolytics:AR+BP+IM | **-** | **-** | 0.26 (-0.10; 0.62) | Very low^1^ | 0.26 (-0.10; 0.62) | Very low^7^ |
| Anxiolytics:BP+IM | **-** | **-** | 0.54 (-0.18; 1.26) | Low^3^ | 0.54 (-0.18; 1.26) | Very low^7^ |
| Anxiolytics:Control group | 0.15 (-0.12; 0.41) | Moderate | **-** | **-** | 0.15 (-0.12; 0.41) | Low^6,7^ |
| Anxiolytics:Lay foot manipulation | **-** | **-** | 0.22 (-0.25; 0.69) | Moderate^5^ | 0.22 (-0.25; 0.69) | Low^7^ |
| AR+BP:AR+BP+IM | **-** | **-** | 0.11 (-0.30; 0.53) | Very low^2^ | 0.11 (-0.30; 0.53) | Very low^7^ |
| AR+BP:BP+IM | **-** | **-** | 0.39 (-0.35; 1.14) | Very low^1^ | 0.39 (-0.35; 1.14) | Very low^7^ |
| AR+BP:Control group | -0.00 (-0.34; 0.33) | Very low | **-** | **-** | 0 (-0.34; 0.33) | Very low^6,7^ |
| AR+BP:Lay foot manipulation | **-** | **-** | 0.07 (-0.45; 0.58) | Very low^1^ | 0.07 (-0.45; 0.58) | Very low^7^ |
| AR+BP+IM:BP+IM | **-** | **-** | 0.28 (-0.43; 0.99) | Very low^1^ | 0.28 (-0.43; 0.99) | Very low^7^ |
| AR+BP+IM:Control group | -0.12 (-0.36; 0.13) | Very low | **-** | **-** | -0.12 (-0.36; 0.13) | Very low^6,7^ |
| AR+BP+IM:Lay foot manipulation | **-** | **-** | -0.05 (-0.50; 0.41) | Very low^1^ | -0.05 (-0.50; 0.41) | Very low^7^ |
| BP+IM:Control group | -0.40 (-1.06; 0.27) | Low |  |  | -0.4 (-1.06; 0.27) | Very low^6,7^ |
| BP+IM:Lay foot manipulation | **-** | **-** | -0.32 (-1.10; 0.45) | Low^3^ | -0.32 (-1.10; 0.45) | Very low^7^ |
| Lay foot manipulation:Control group | -0.07 (-0.46; 0.32) | Moderate | **-** | **-** | -0.07 (-0.46; 0.32) | Low^6,7^ |

Note: Activity rehabilitation+Behavioral psychoeducational = AR+BP; Activity rehabilitation+Behavioral psychoeducational+Integrative medicine = AR+BP+IM; Behavioral psychoeducational+Integrative medicine = BP+IM.

^1^ The lower confidence rating of the two direct comparisons is very low,

^2^ The confidence ratings for both direct comparisons are very low,

^3^ The lower confidence rating of the two direct comparisons is low,

^4^ The confidence ratings for both direct comparisons are low,

^5^ The confidence ratings for both direct comparisons are moderate,

^6^ The contribution of the direct evidence to the network estimate is much greater than that of the indirect evidence,

^7^ Imprecise (95% CI includes SMD of 0).

**Outcome 3: Exercise Capacity**

| **Comparison** | **Direct evidence** | | **Indirect evidence** | | **Network Meta-analysis** | |
| --- | --- | --- | --- | --- | --- | --- |
|  | **MD (95%CI)** | **Certainty of evidence** | **MD (95%CI)** | **Certainty of evidence** | **MD (95%CI)** | **Certainty of evidence** |
| Activity rehabilitation:Acupressure or Reflexology | **-** | **-** | 8.77 (-133.61; 151.15) | Very low^1^ | 8.77 (-133.61; 151.15) | Very low^4^ |
| Activity rehabilitation:AR+BP | **-** | **-** | 48.80 (-193.27; 290.87) | Very low^1^ | 48.80 (-193.27; 290.87) | Very low^4^ |
| Activity rehabilitation:Compressed air | **-** | **-** | 57.01 (-89.09; 203.12) | Very low^1^ | 57.01 (-89.09; 203.12) | Very low^4^ |
| Activity rehabilitation:Control group | 56.10 (-16.96; 129.16) | Very low | **-** | **-** | 56.10 (-16.96; 129.16) | Very low^3,4^ |
| Activity rehabilitation:Opioids | **-** | **-** | 49.49 (-53.78; 152.77) | Very low^1^ | 49.49 (-53.78; 152.77) | Very low^4^ |
| Acupressure or Reflexology:AR+BP | **-** | **-** | 40.03 (-221.11; 301.17) | Low^2^ | 40.03 (-221.11; 301.17) | Very low^4^ |
| Acupressure or Reflexology:Compressed air | **-** | **-** | 48.24 (-127.67; 224.16) | Low^2^ | 48.24 (-127.67; 224.16) | Very low^4^ |
| Acupressure or Reflexology:Control group | 47.33 (-74.88; 169.54) | Low | **-** | **-** | 47.33 (-74.88; 169.54) | Very low^3,4^ |
| Acupressure or Reflexology:Opioids | **-** | **-** | 40.72 (-101.63; 183.08) | Low^2^ | 40.72 (-101.63; 183.08) | Very low^4^ |
| AR+BP:Compressed air | **-** | **-** | 8.21 (-254.98; 271.41) | Low^2^ | 8.21 (-254.98; 271.41) | Very low^4^ |
| AR+BP:Control group | 7.30 (-223.48; 238.08) | Low | **-** | **-** | 7.3 (-223.48; 238.08) | Very low^3,4^ |
| AR+BP:Opioids | **-** | **-** | 0.69 (-241.36; 242.75) | Low^2^ | 0.69 (-241.36; 242.75) | Very low^4^ |
| Compressed air:Control group | -0.91 (-127.45; 125.62) | Low | **-** | **-** | -0.91 (-127.45; 125.62) | Very low^3,4^ |
| Compressed air:Opioids | **-** | **-** | -7.52 (-153.60; 138.56) | Low^2^ | -7.52 (-153.60; 138.56) | Very low^4^ |
| Opioids:Control group | 6.61 ( -66.39; 79.61) | Low | **-** | **-** | 6.61 ( -66.39; 79.61) | Very low^3,4^ |

Note: Activity rehabilitation+Behavioral psychoeducational = AR+BP.

^1^ The lower confidence rating of the two direct comparisons is very low,

^2^ The confidence ratings for both direct comparisons are low,

^3^ The contribution of the direct evidence to the network estimate is much greater than that of the indirect evidence,

^4^ Imprecise (95% CI includes MD of 0)

**Outcome 4: Health-related Quality of Life**

| **Comparison** | **Direct evidence** | | **Indirect evidence** | | **Network Meta-analysis** | |
| --- | --- | --- | --- | --- | --- | --- |
|  | **SMD (95%CI)** | **Certainty of evidence** | **SMD (95%CI)** | **Certainty of evidence** | **SMD (95%CI)** | **Certainty of evidence** |
| Activity rehabilitation:Acupressure or Reflexology | **-** | **-** | -1.33 (-2.99; 0.32) | Very low^1^ | -1.33 (-2.99; 0.32) | Very low^7^ |
| Activity rehabilitation:Airflow | **-** | **-** | -0.85 (-2.96; 1.26) | Very low^1^ | -0.85 (-2.96; 1.26) | Very low^7^ |
| Activity rehabilitation:AR+BP | **-** | **-** | -0.05 (-1.70; 1.60) | Very low^1^ | -0.05 (-1.70; 1.60) | Very low^7^ |
| Activity rehabilitation:AR+BP+IM | **-** | **-** | -0.09 (-1.73; 1.55) | Very low^1^ | -0.09 (-1.73; 1.55) | Very low^7^ |
| Activity rehabilitation:Behavioral psychoeducational | **-** | **-** | 0.07 (-1.57; 1.72) | Very low^1^ | 0.07 (-1.57; 1.72) | Very low^7^ |
| Activity rehabilitation:Cannabidiol | **-** | **-** | 0.04 (-2.05; 2.13) | Very low^1^ | 0.04 (-2.05; 2.13) | Very low^7^ |
| Activity rehabilitation:Control group | 0.22 (-0.76; 1.20) | Very low | **-** | **-** | 0.22 (-0.76; 1.20) | Very low^6,7^ |
| Activity rehabilitation:Lay foot manipulation | **-** | **-** | -0.51 (-2.48; 1.47) | Very low^1^ | -0.51 (-2.48; 1.47) | Very low^7^ |
| Acupressure or Reflexology:Airflow | **-** | **-** | 0.48 (-1.81; 2.78) | Low^3^ | 0.48 (-1.81; 2.78) | Very low^7^ |
| Acupressure or Reflexology:AR+BP | **-** | **-** | 1.28 (-0.60; 3.16) | Moderate^5^ | 1.28 (-0.60; 3.16) | Low^7^ |
| Acupressure or Reflexology:AR+BP+IM | **-** | **-** | 1.24 (-0.63; 3.12) | Moderate^5^ | 1.24 (-0.63; 3.12) | Low^7^ |
| Acupressure or Reflexology:Behavioral psychoeducational | **-** | **-** | 1.41 (-0.47; 3.28) | Low^3^ | 1.41 (-0.47; 3.28) | Very low^7^ |
| Acupressure or Reflexology:Cannabidiol | **-** | **-** | 1.38 (-0.90; 3.65) | Moderate^5^ | 1.38 (-0.90; 3.65) | Low^7^ |
| Acupressure or Reflexology:Control group | 1.55 (0.22; 2.88) | Moderate | **-** | **-** | 1.55 (0.22; 2.88) | Moderate^6^ |
| Acupressure or Reflexology:Lay foot manipulation | 0.07 (-1.75; 1.89) | Low | 6.58 (1.55; 11.60) | Low^3^ | 0.83 (-0.89; 2.54) | Very low^6,7^ |
| Airflow:AR+BP | **-** | **-** | 0.80 (-1.49; 3.09) | Low^3^ | 0.80 (-1.49; 3.09) | Very low^7^ |
| Airflow:AR+BP+IM | **-** | **-** | 0.76 (-1.52; 3.04) | Low^3^ | 0.76 (-1.52; 3.04) | Very low^7^ |
| Airflow:Behavioral psychoeducational | **-** | **-** | 0.92 (-1.36; 3.21) | Low^4^ | 0.92 (-1.36; 3.21) | Very low^7^ |
| Airflow:Cannabidiol | **-** | **-** | 0.89 (-1.73; 3.51) | Low^3^ | 0.89 (-1.73; 3.51) | Very low^7^ |
| Airflow:Control group | 1.07 (-0.80; 2.93) | Low | **-** | **-** | 1.07 (-0.80; 2.93) | Very low^6,7^ |
| Airflow:Lay foot manipulation | **-** | **-** | 0.34 (-2.19; 2.88) | Low^4^ | 0.34 (-2.19; 2.88) | Very low^7^ |
| AR+BP:AR+BP+IM | **-** | **-** | -0.04 (-1.91; 1.83) | Moderate^5^ | -0.04 (-1.91; 1.83) | Low^7^ |
| AR+BP:Behavioral psychoeducational | **-** | **-** | 0.13 (-1.75; 2.00) | Low^3^ | 0.13 (-1.75; 2.00) | Very low^7^ |
| AR+BP:Cannabidiol | **-** | **-** | 0.09 (-2.18; 2.37) | Moderate^5^ | 0.09 (-2.18; 2.37) | Low^7^ |
| AR+BP:Control group | 0.27 (-1.06; 1.60) | Moderate | **-** | **-** | 0.27 (-1.06; 1.60) | Low^6,7^ |
| AR+BP:Lay foot manipulation | **-** | **-** | -0.45 (-2.62; 1.72) | Low^3^ | -0.45 (-2.62; 1.72) | Very low^7^ |
| AR+BP+IM:Behavioral psychoeducational | **-** | **-** | 0.17 (-1.70; 2.03) | Low^3^ | 0.17 (-1.70; 2.03) | Very low^7^ |
| AR+BP+IM:Cannabidiol | **-** | **-** | 0.13 (-2.13; 2.40) | Moderate^5^ | 0.13 (-2.13; 2.40) | Low^7^ |
| AR+BP+IM:Control group | 0.31 (-1.01; 1.62) | Moderate | **-** | **-** | 0.31 (-1.01; 1.62) | Low^6,7^ |
| AR+BP+IM:Lay foot manipulation | **-** | **-** | -0.42 (-2.58; 1.75) | Low^3^ | -0.42 (-2.58; 1.75) | Very low^7^ |
| Behavioral psychoeducational:Cannabidiol | **-** | **-** | -0.03 (-2.30; 2.24) | Low^3^ | -0.03 (-2.30; 2.24) | Very low^7^ |
| Behavioral psychoeducational:Control group | 0.14 (-1.18; 1.46) | Low | **-** | **-** | 0.14 (-1.18; 1.46) | Very low^6,7^ |
| Behavioral psychoeducational:Lay foot manipulation | **-** | **-** | -0.58 (-2.75; 1.58) | Low^4^ | -0.58 (-2.75; 1.58) | Very low^7^ |
| Cannabidiol:Control group | 0.17 (-1.67; 2.02) | Moderate | **-** | **-** | 0.17 (-1.67; 2.02) | Low^6,7^ |
| Cannabidiol:Lay foot manipulation | **-** | **-** | -0.55 (-3.07; 1.97) | Low^3^ | -0.55 (-3.07; 1.97) | Very low^7^ |
| Lay foot manipulation:Control group | -0.04 (-1.86; 1.79) | Low | 6.45 (1.44; 11.47) | Low^3^ | 0.72 (-0.99; 2.44) | Very low^6,7^ |

Note: Activity rehabilitation+Behavioral psychoeducational = AR+BP; Activity rehabilitation+Behavioral psychoeducational+Integrative medicine = AR+BP+IM; Behavioral psychoeducational+Integrative medicine = BP+IM.

^1^ The lower confidence rating of the two direct comparisons is very low,

^2^ The confidence ratings for both direct comparisons are very low,

^3^ The lower confidence rating of the two direct comparisons is low,

^4^ The confidence ratings for both direct comparisons are low,

^5^ The confidence ratings for both direct comparisons are moderate,

^6^ The contribution of the direct evidence to the network estimate is much greater than that of the indirect evidence,

^7^ Imprecise (95% CI includes SMD of 0)

# Appendix 8. Funnel plots for all outcomes


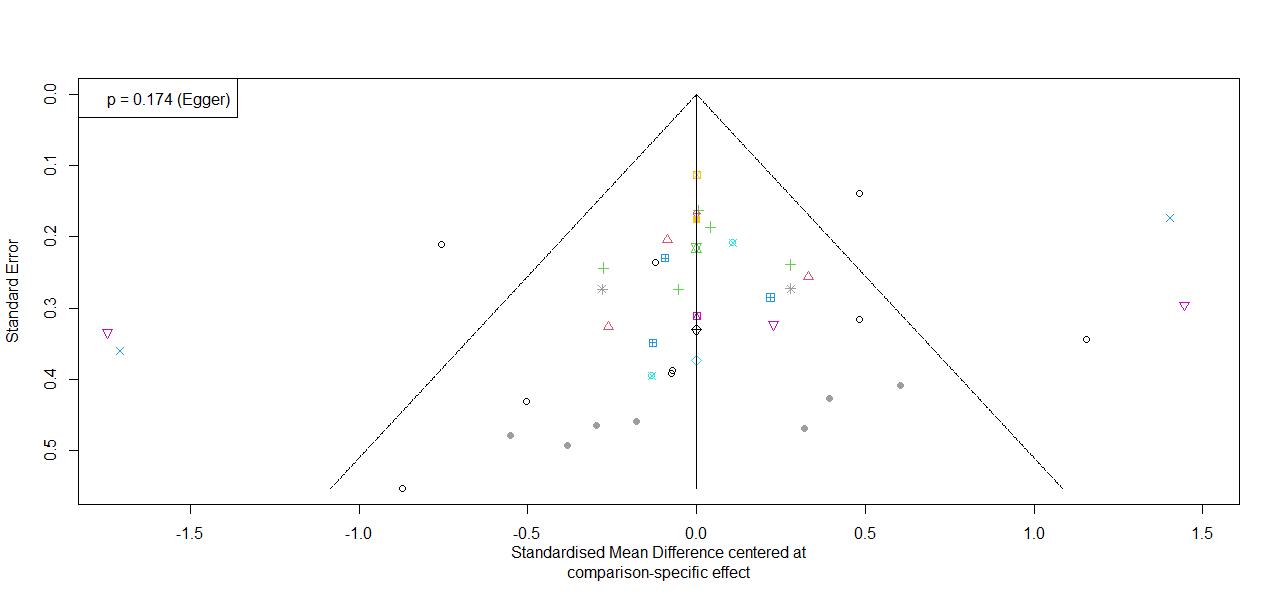


**Fig. 8.1. Funnel plot for Dyspnea Severity**


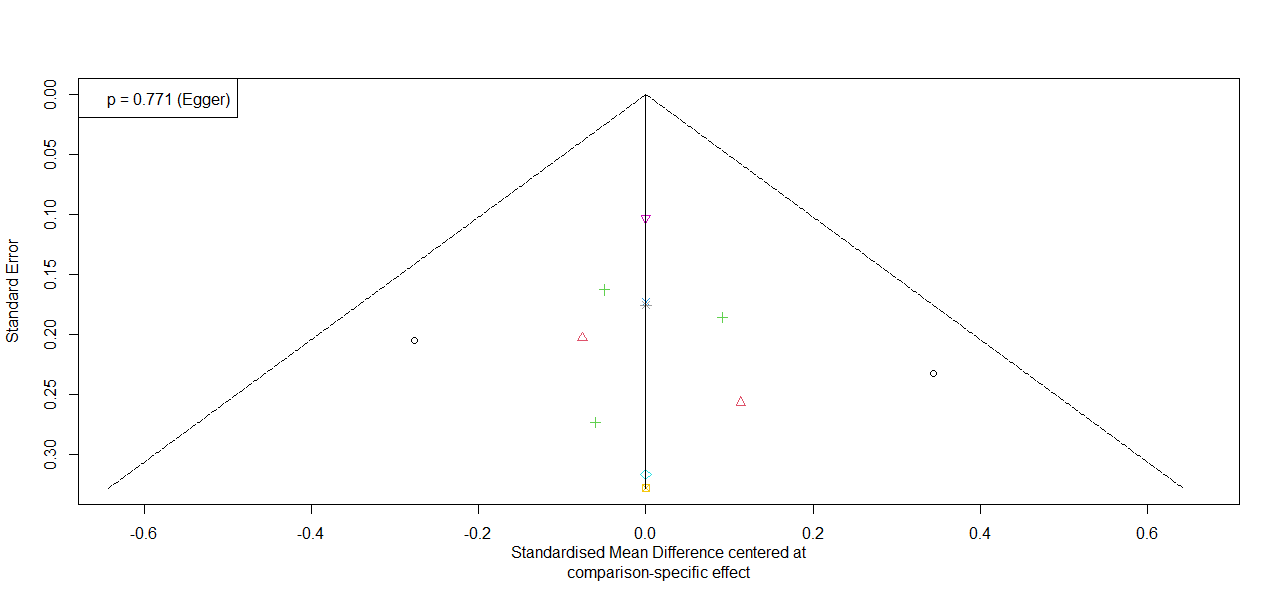


**Fig. 8.2. Funnel plot for Anxiety**


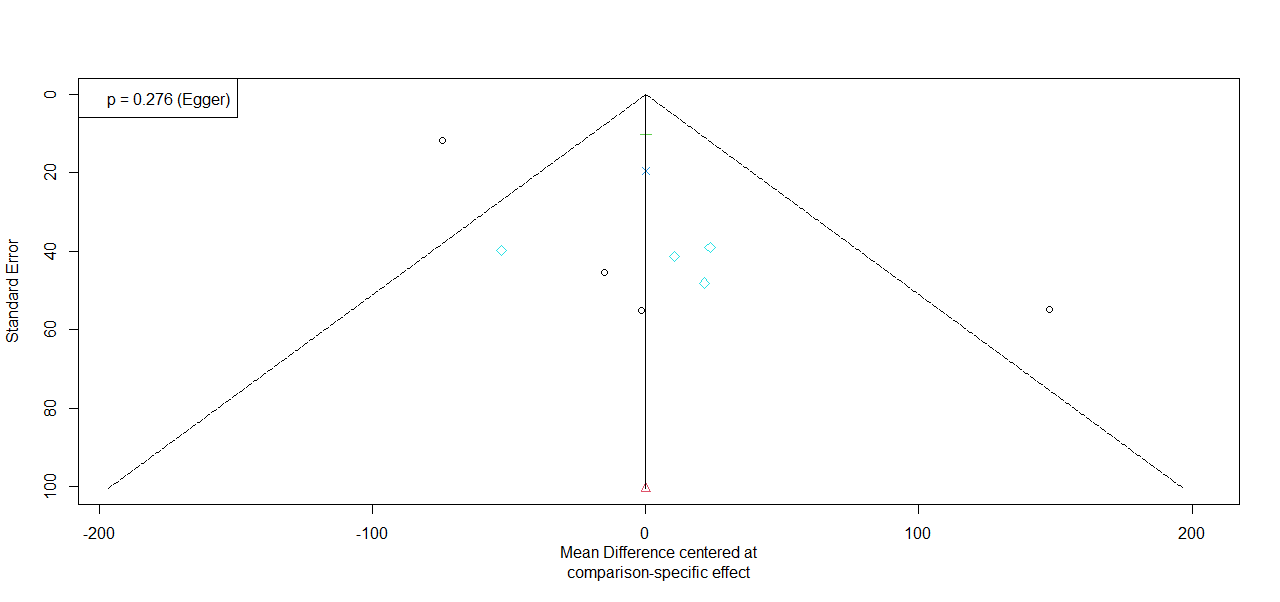


**Fig. 8.3. Funnel plot for Exercise Capacity**


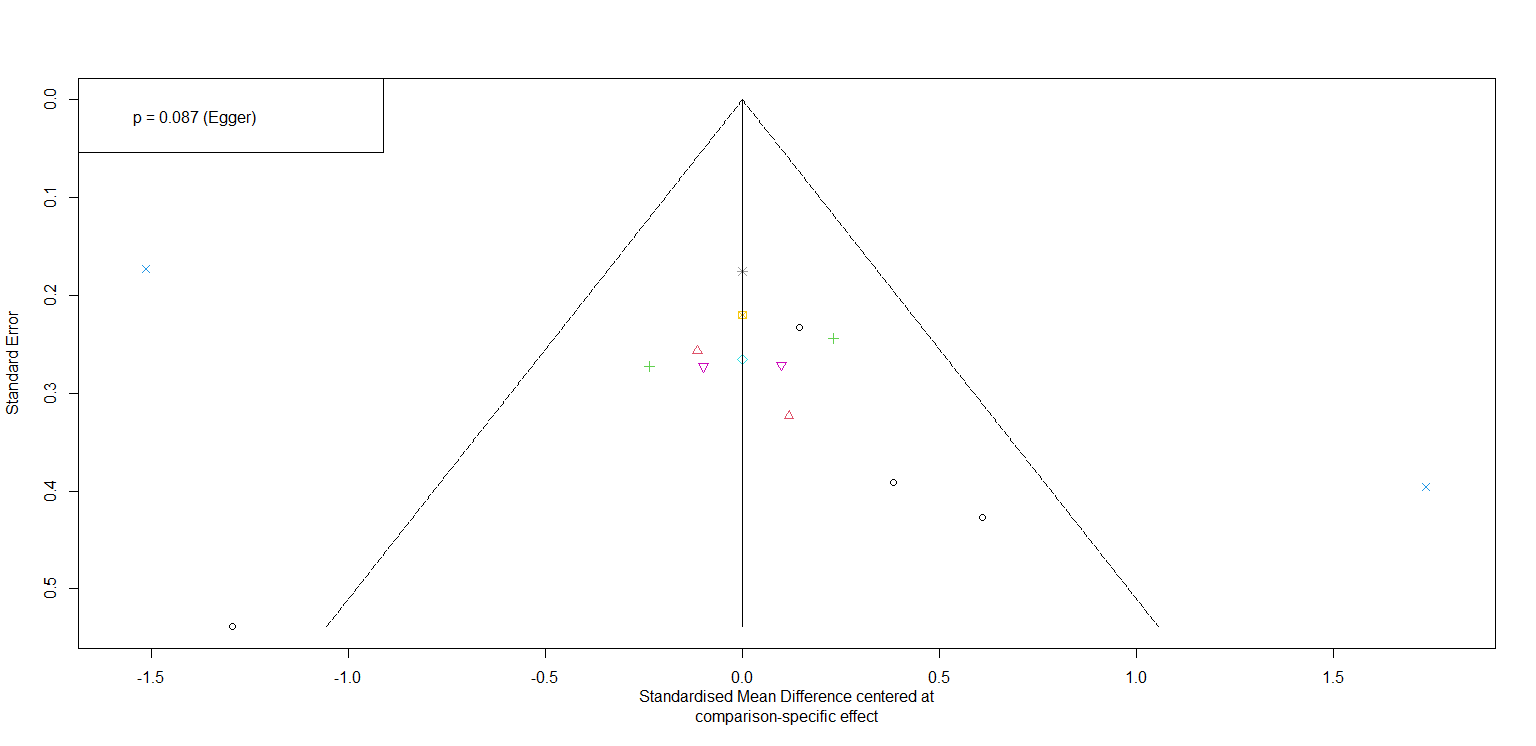


**Fig. 8.4. Funnel plot for Health-related Quality of Life**

# Appendix 9. Sensitivity analysis

**9.1 Outcome 1: Dyspnea Severity**

10 studies at high risk of bias of overall domains (Bade 2021; Chan 2011; Henke 2014; Farquhar 2014; Yorke 2015; Kocatepe 2021; Moore 2002; Bruera 1993; Molassiotis 2014; Yorke 2023; Xu 2023; Jastrzebski 2015) were excluded.

| **Comparison** | **Base data** | | **Overall domains** | |
| --- | --- | --- | --- | --- |
|  | **No.** | **SMD (95% CI)** | **No.** | **SMD (95% CI)** |
| Activity rehabilitation:CG | 9 | -0.42 (-0.90; 0.06) | 5 | -0.62 (-1.29; 0.05) |
| Acupressure/Reflexology:CG | 2 | -1.04 (-2.02; -0.06) | 2 | -1.06 (-2.09; -0.02) |
| Acupuncture:CG | 1 | 0.58 (-0.89; 2.05) | 1 | 0.58 (-0.97; 2.12) |
| Airflow:CG | 3 | -0.81 (-1.63; 0.01) | 2 | 0.03 (-1.02; 1.09) |
| Anxiolytics:CG | 1 | 0.13 (-1.17; 1.42) | 1 | 0.13 (-1.25; 1.50) |
| AR+BP:CG | 3 | -0.30 (-1.10; 0.50) | 1 | 0.03 (-1.42; 1.48) |
| AR+BP+IM:CG | 5 | -0.28 (-0.88; 0.33) | 2 | -0.12 (-1.13; 0.89) |
| Behavioral psychoeducational:CG | 2 | -0.18 (-1.16; 0.80) | 1 | 0.10 (-1.36; 1.56) |
| Bilevel ventilation:CG | 1 | -0.42 (-1.74; 0.89) | 1 | -0.42 (-1.82; 0.98) |
| BP+IM:CG | 1 | 0.54 (-0.89; 1.97) | 1 | 0.54 (-0.97; 2.04) |
| Cannabidiol:CG | 1 | -0.01 (-1.36; 1.33) | 1 | -0.01 (-1.44; 1.41) |
| Compressed air:CG | 3 | 0.29 (-0.52; 1.10) | 3 | 0.29 (-0.56; 1.14) |
| Corticosteroids:CG | 2 | -0.11 (-1.10; 0.89) | 2 | -0.11 (-1.16; 0.94) |
| HFNC:CG | 1 | -1.91 (-3.32; -0.49) | 1 | -1.91 (-3.40; -0.42) |
| Lay foot manipulation:CG | 1 | -0,34 (-1.59; 0.90) | 1 | -0.35 (-1.67; 0.97) |
| Opioids:CG | 7 | -0.49 (-1.08; 0.10) | 6 | -0.44 (-1.11; 0.22) |

No.: numbers of studies included. SMD: Standardized Mean Difference. CI: confident interval. Activity rehabilitation + Behavioral psychoeducational = AR+BP. Activity rehabilitation + Behavioral psychoeducational + Integrative medicine = AR+BP+IM. Behavioral psychoeducational + Integrative medicine = BP+IM. CG = Control group

**9.2 Outcome 2: Anxiety**

3 studies at high risk of bias of overall domains (Chan 2011; Farquhar 2014; Yorke 2023) were excluded.

| **Comparison** | **Base data** | | | **Overall domains** |
| --- | --- | --- | --- | --- |
|  | **No.** | **SMD (95% CI)** | **No.** | **SMD (95% CI)** |
| Activity rehabilitation:CG | 2 | -0.64 (-0.97; -0.32) | 2 | -0.62 (-1.23; -0.01) |
| Acupressure or Reflexology:CG | 1 | 0.12 (-0.27; 0.50) | 1 | 0.12 (-0.70; 0.93) |
| Airflow:CG | 1 | 0.13 (-0.52; 0.77) | 1 | 0.13 (-0.84; 1.10) |
| Anxiolytics:CG | 1 | 0.15 (-0.12; 0.41) | 1 | 0.15 (-0.63; 0.92) |
| AR+BP:CG | 2 | 0.00 (-0.34; 0.33) | 1 | 0.11 (-0.79; 1.01) |
| AR+BP+IM:CG | 3 | -0.12 (-0.36; 0.13) | 1 | -0.02 (-0.85; 0.80) |
| BP+IM:CG | 1 | -0.40 (-1.06; 0.27) | 1 | -0.40 (-1.38; 0.59) |
| Lay foot manipulation:CG | 1 | -0.07 (-0.46; 0.32) | 1 | -0.07 (-0.89; 0.75) |

No.: numbers of studies included. SMD: Standardized Mean Difference. CI: confident interval. Activity rehabilitation + Behavioral psychoeducational = AR+BP. Activity rehabilitation + Behavioral psychoeducational + Integrative medicine = AR+BP+IM. Behavioral psychoeducational + Integrative medicine = BP+IM. CG = Control group

**9.3 Outcome 3: Exercise Capacity**

3 studies at high risk of bias of overall domains (Henke 2014; Jastrzebski 2015; Xu 2023) were excluded.

| **Comparison** | **Base data** | | **Overall domains** | |
| --- | --- | --- | --- | --- |
|  | **No.** | **MD (95% CI)** | **No.** | **MD (95% CI)** |
| Activity rehabilitation:CG | 4 | 56.10 (-16.96; 129.16) | 1 | 41.00 (-48.13; 130.13) |
| Acupressure or Reflexology:CG | 1 | **47.33 (-74.88; 169.54)** | 1 | **47.33 (27.40; 67.26)** |
| AR+BP:CG | 1 | 7.30 (-223.48; 238.08) | 1 | 7.30 (-189.48; 204.08) |
| Compressed air:CG | 1 | -0.91 (-127.45; 125.62) | 1 | -0.91 (-39.29; 37.46) |
| Opioids:CG | 4 | 6.61 (-66.39; 79.61) | 4 | 5.53 (-35.28; 46.34) |

No.: numbers of studies included. SMD: Standardized Mean Difference. CI: confident interval. Activity rehabilitation + Behavioral psychoeducational = AR+BP. Activity rehabilitation + Behavioral psychoeducational + Integrative medicine = AR+BP+IM. Behavioral psychoeducational + Integrative medicine = BP+IM. CG = Control group

**9.4 Outcome 4: Health-related Quality of Life**

6 studies at high risk of bias of overall domains (Henke 2014; Farquhar 2014; Moore 2002; Bade 2021; Kocatepe 2021; Jastrzebski 2015) were excluded.

| **Comparison** | **Base data** | | **Overall domains** | |
| --- | --- | --- | --- | --- |
|  | **No.** | **SMD (95% CI)** | **No.** | **SMD (95% CI)** |
| Activity rehabilitation:CG | 4 | 0.22 (-0.76; 1.20) | 2 | 0.59 (-1.82; 2.99) |
| Acupressure or Reflexology:CG | 2 | **1.55 (0.22; 2.88)** | 2 | **1.63 (-0.77; 4.02)** |
| Airflow:CG | 1 | 1.07 (-0.80; 2.93) | No studies | |
| AR+BP:CG | 2 | 0.27 (-1.06; 1.60) | 1 | 0.16 (-3.22; 3.53) |
| AR+BP+IM:CG | 2 | 0.31 (-1.01; 1.62) | 1 | 0.54 (-2.83; 3.91) |
| Behavioral psychoeducational:CG | 2 | 0.14 (-1.18; 1.46) | 1 | 0.04 (-3.33; 3.42) |
| Cannabidiol:CG | 1 | 0.17 (-1.67; 2.02) | 1 | 0.17 (-3.19; 3.54) |
| Lay foot manipulation:CG | 1 | 0.72 (-0.99; 2.44) | 1 | 0.76 (-2.38; 3.90) |

No.: numbers of studies included. SMD: Standardized Mean Difference. CI: confident interval. Activity rehabilitation + Behavioral psychoeducational = AR+BP. Activity rehabilitation + Behavioral psychoeducational + Integrative medicine = AR+BP+IM. Behavioral psychoeducational + Integrative medicine = BP+IM. CG = Control group

# Appendix 10. Risk of bias of each RCT

**Table 10.1 Risk of bias assessment of included RCT (n=32)**


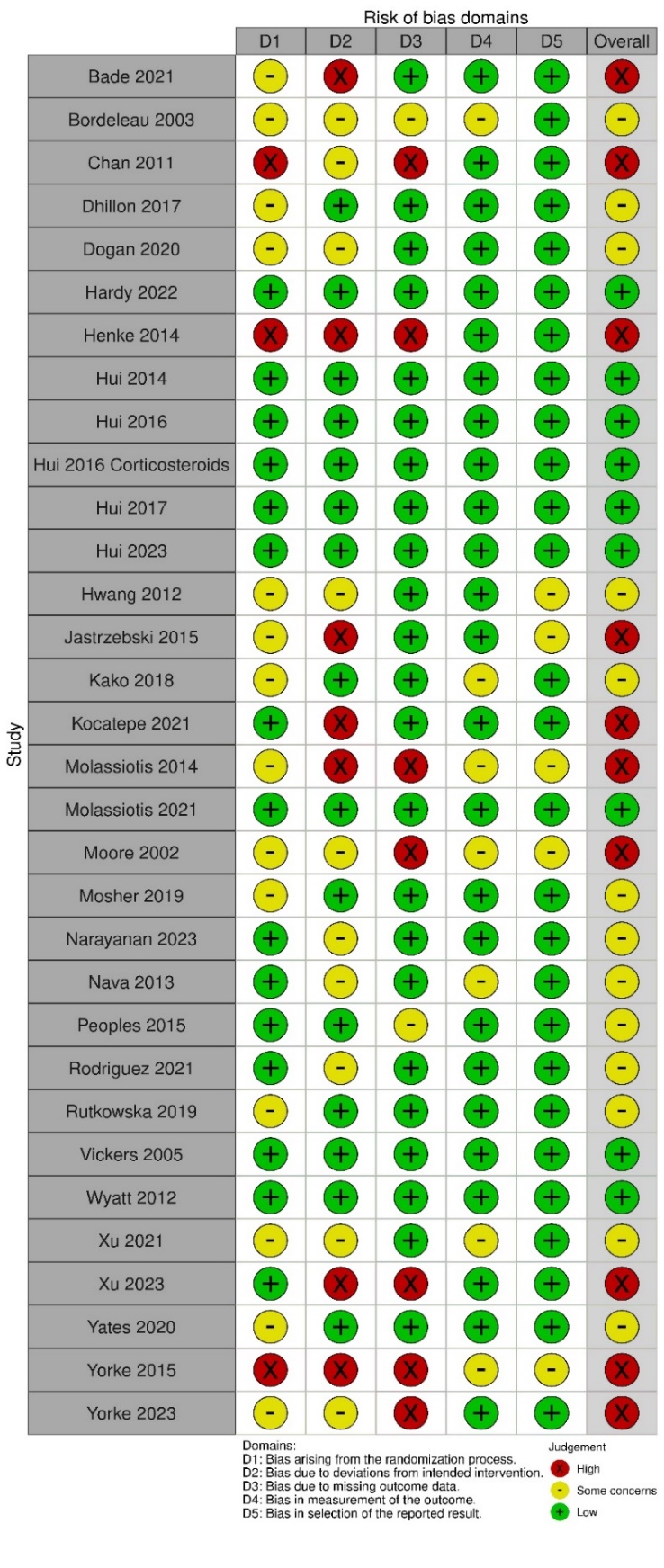


**Figure 10.1: Risk of Bias Summary plots for RCTs**

**Table 10.2 Risk of bias assessment of included crossover RCT (n=10)**


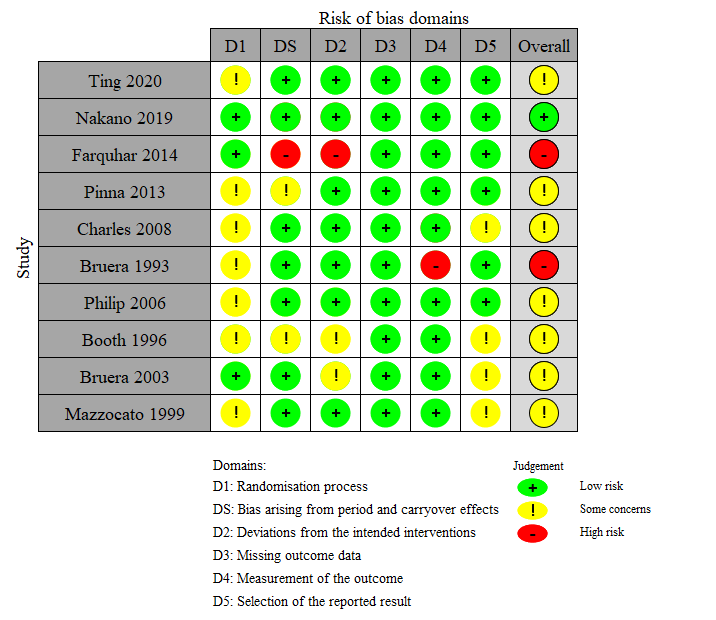


**Figure 10.2 Risk of Bias Summary plots for Crossover RCTs**
